# Markers of resilience to stony coral tissue loss disease and putative probiotic functions in the microbiomes of the coral, *Orbicella faveolata*

**DOI:** 10.1093/ismeco/ycag278

**Published:** 2026-10-01

**Authors:** Cynthia C Becker, Allison R Cauvin, Karen L Neely, Caroline E Dennison, Andrew C Baker, Brian K Walker, Julie L Meyer

**Affiliations:** Department of Soil, Water, and Ecosystem Sciences, Genetics Institute, Institute of Food and Agricultural Sciences, University of Florida, Gainesville, FL 32611, United States; School of Natural Resources and Environment, University of Florida, Gainesville, FL 32611, United States; National Coral Reef Institute, Nova Southeastern University, Ft. Lauderdale, FL 33328, United States; Department of Marine Biology and Ecology, Rosenstiel School of Marine, Atmospheric, and Earth Science, University of Miami, Miami, FL 33149, United States; Department of Marine Biology and Ecology, Rosenstiel School of Marine, Atmospheric, and Earth Science, University of Miami, Miami, FL 33149, United States; National Coral Reef Institute, Nova Southeastern University, Ft. Lauderdale, FL 33328, United States; Department of Soil, Water, and Ecosystem Sciences, Genetics Institute, Institute of Food and Agricultural Sciences, University of Florida, Gainesville, FL 32611, United States

**Keywords:** holobiont, coral microbiome, intraspecies variation, disease resistance, antimicrobial resistance, metagenome

## Abstract

Stony coral tissue loss disease (SCTLD) is widespread within the Caribbean and affects at least 22 species of reef-building coral. Bacteria have been implicated in the etiology of SCTLD, but the community of bacteria and archaea may also contribute to SCTLD resistance. To identify potential mechanisms through which microbes contribute to SCTLD resistance, we sequenced metagenomes from 41 colonies of the threatened coral, *Orbicella faveolata*, in the lower Florida Keys. All colonies were fate-tracked for 4 to 5 years, treated with amoxicillin if needed to prevent SCTLD-related mortality, and categorized into 4 health status categories following the monitoring period: never diseased (20%), diseased before sampling but recovered (10%), apparently healthy during sampling but eventually diseased (22%), and diseased (49%). Within the coral microbiome, diseased and yet-to-be diseased colonies exhibited higher variability in functional genes. In contrast, corals that remained unaffected or recovered had less variable microbiomes with greater abundances of vitamin and antibiotic biosynthesis, secretion system, and quorum-sensing genes that may support host health and resilience to pathogens. Despite repeated antibiotic treatments on some colonies, there was no effect on the diversity of beta-lactamases, antibiotic resistance genes that may confer amoxicillin resistance. Additional potentially probiotic gene clusters for the production of antimicrobial and bioactive compounds were present in many colonies regardless of fate. Taken together, we find significant putative probiotic functions in the coral microbiome to armor host *O. faveolata* corals against SCTLD infection, which may underpin intraspecific variation in stony coral tissue loss disease resilience and susceptibility.

## Introduction

Stony coral tissue loss disease (SCTLD) is a contagious disease affecting at least 22 species of reef-building corals in Florida and the Caribbean [1]. The outbreak began in Florida in 2014 and resulted in significant losses to coral colony density and coral cover [2, 3], near extirpation of some highly susceptible species [4], and decline in ecosystem functions such as reef accretion [5]. While some corals are highly susceptible to this disease, others show varying susceptibility and resist infection for years [reviewed by 1, 6]. The mountainous star coral, *Orbicella faveolata,* which is listed as threatened under the US Endangered Species Act and as endangered by the International Union for Conservation of Nature (IUCN), is a bouldering, reef-building coral historically common in the Caribbean with moderate susceptibility to SCTLD relative to other corals [6]. Within a diseased reef, some *O. faveolata* colonies seem resistant and do not develop lesions even over years of exposure [7], while others nearby quickly and repeatedly develop numerous lesions [8, 9]. The underlying mechanisms contributing to SCTLD resistance by some colonies remain an open area of research.

Resistance to SCTLD may be due to any number of coral traits within the holobiont. The coral holobiont consists of the coral host and its symbiotic dinoflagellate algae (Symbiodiniaceae), bacteria, archaea, viruses, and fungi [10]. Here, we focus on the role of the microbial communities within the holobiont and their potential for supporting host disease resistance and susceptibility. We define the coral microbiome as the prokaryotic component of the holobiont (bacteria, archaea), which is known to support coral host health as well as cause disease [11, 12]. This microbiome confers benefits to the holobiont by cycling nitrogen [13], providing essential vitamins [14], and providing probiotic benefits like antibiotic defenses [15, 16]. However, the microbiome may also cause diseases [17, 18]. While the causative agent of SCTLD remains elusive, the lesions harbor potentially pathogenic genera and known pathogens like *Vibrio coralliilyticus* [19–25]. Therefore, investigating the microbiome within the host offers a promising avenue to better understand SCTLD resistance.

While 16S rRNA gene sequencing provides insight into bacteria that may play a role in the disease, this approach lacks information on functional genes that would inform disease etiology. For example, many studies associate *Vibrio* with SCTLD [19, 20, 23, 25], but healthy coral hosts often harbor *Vibrio* bacteria that play a beneficial role in nitrogen acquisition [26]. Additionally, 16S rRNA gene sequencing is often unable to differentiate between pathogenic and nonpathogenic *Vibrio* species [27, 28]. Therefore, we focused on metagenomics-based approaches, which have previously shed light on SCTLD pathogenesis [29–31]. Extending this previous research, we apply metagenomics-based methods to identify microbial markers that may shape host resistance to infection.

Incorporating symbiont composition into resistance analyses is important for understanding SCTLD dynamics. Zooxanthellate scleractinian corals form an obligate symbiosis with photosynthetic dinoflagellates in the Family Symbiodiniaceae [32, 33], which can be independently susceptible to diseases that affect their mutualism with the coral host. In the case of SCTLD, histopathology and field observations suggest involvement of the Symbiodiniaceae in affected corals [34]. It is hypothesized that disruption (and eventual degradation) of the Symbiodiniaceae is the first sign of disease, which eventually leads to SCTLD lesions [34]. *In situ* observations support this idea because SCTLD quiesces upon coral paling or bleaching, a sign of the coral host expelling its algal symbionts [8, 35–37]. Along with Symbiodiniaceae density, symbiont species may affect SCTLD susceptibility [38].

To identify microbial signatures of disease resistance, we sequenced metagenomes from apparently healthy tissue of 41 fate-tracked *O. faveolata* coral colonies in the lower Florida Keys (Fig. 1, Table S1). These corals had known health histories before and after sampling, and those with SCTLD lesions were treated with topical amoxicillin paste to preserve tissue and prevent mortality [8, 9, 39]. We hypothesized that colony fate, coral disease condition, and underlying symbiont community would influence the functional potential of the microbiome. To identify markers of disease resilience within the coral microbiome, we compared corals of distinct SCTLD fates. Additionally, given the variable disease susceptibility and repeated treatments with amoxicillin, we hypothesized that the coral microbiome harbored diverse antibiotic resistance genes and other biosynthetic gene clusters that produce antimicrobial compounds.

**Figure 1:**
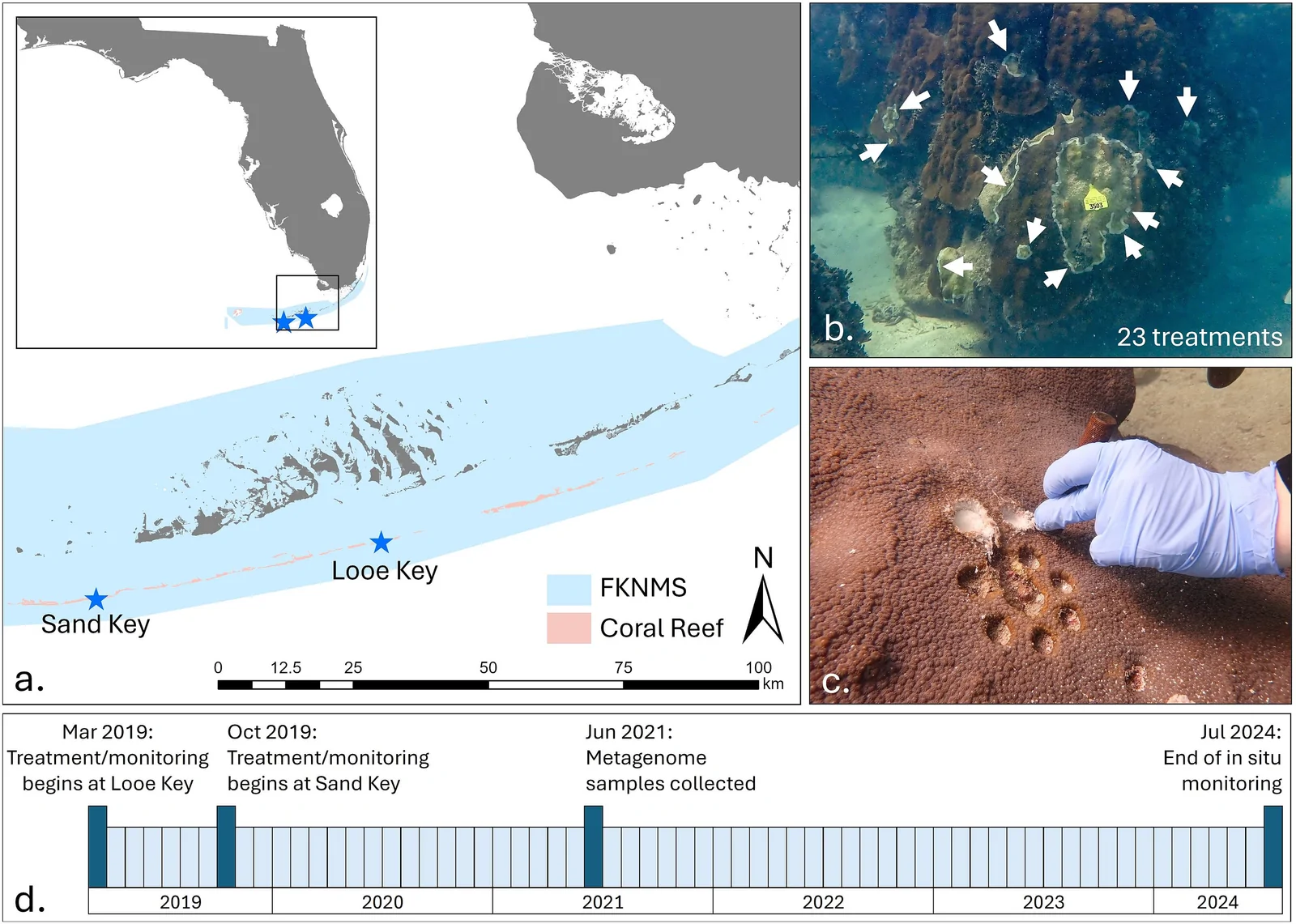
(a) Location of the two sampling sites (Sand Key and Looe Key) within the lower Florida Keys and the Florida Keys National Marine Sanctuary (FKNMS) monitored from 2019 to 2024. (b) Example of an *O. faveolata* with numerous amoxicillin treatments (arrows; 23 were recorded for this coral on this date, but not all are visible in this photo). Note that treatments are highly variable in size and are also spatially dispersed across the colony, with some tissue isolates not having any treatments and some having many. (c) Collection of a sample from an *O. faveolata* colony using a 12 mm leather punch. Adjacent cores were collected for other collaborative projects. (d) Timeline indicating the beginning of SCTLD treatments and monitoring at each sampling site, sampling of coral colonies for bacterial metagenomics, and the end of monitoring and treatments in the field. Monitoring events, treatment applications, and resultant colony fate are summarized in Table S1 as monitoring timeframe differed among colonies.

## Materials and methods

### Study area and collection

Core samples were taken from 41 *O. faveolata* colonies at 2 reef sites in the lower Florida Keys: Looe Key and Sand Key (Fig. 1a). The selected corals were part of a long-term SCTLD intervention and monitoring project across multiple species and sites [8, 9, 39]. Samples from the selected corals were collected by the SCTLD Resistance Research Consortium (RRC) to measure over a dozen holobiont traits [40]. In-water amoxicillin interventions and fate-tracking were initiated at Looe Key in March 2019 and at Sand Key in October 2019 (Fig. 1d, Table S1). Each site was visited approximately every 2 months through July 2024, and any coral affected by SCTLD was tagged, mapped, photographed, and treated on active disease lesions with a topical amoxicillin paste (see SI Methods). All previously tagged corals were monitored for health status during each visit. Based on the health histories of these corals, *O. faveolata* at both sites were selected to represent a range of disease susceptibility levels for further analysis as part of the RRC [40].

Coral cores were collected via SCUBA on 12–13 June 2021. Separate 12 mm leather punches were used to extract a 1 cm core from apparently healthy tissue on each colony far from active SCTLD lesions (Fig. 1c). Tissue cores were placed into a labeled bag. At the surface, they were preserved in DNA/RNA Shield (Zymo Research, Irvine, CA, USA) and kept on ice. All samples were stored at −80°C until processing.

### Colony characteristics

Following 4 to 5 years of fate-tracking (Table S1), we developed a category of colony fate that incorporated the full health histories of the colonies. Each colony was classified as either: (i) “diseased” (had SCTLD before, during, and after sampling), (ii) “will be diseased” (was healthy at the time of sampling, but developed SCTLD lesions 4–30 months after sampling), (iii) “recovered” (coral had SCTLD before sampling, but not during sampling and at no point after sampling), or (iv) “unaffected” (coral was never observed with SCTLD). Collaborative partners assessed coral host genotypes as described in Klein et al. [41] and Symbiodiniaceae community composition via an actin-based qPCR assay as described in Cunning et al. [42] for each colony (see SI Methods).

### DNA extraction and library preparation

For extraction of prokaryotic DNA, coral cores were thawed on ice, then 1–2 polyps of coral tissue and mucus were scraped from cores using flame-sterilized scalpels and forceps. The tissue was placed in 1 ml of phosphate-buffered saline with ethylenediaminetetraacetic acid as previously described in a coral metagenome extraction protocol [43] and mechanically broken up with a sterile micropestle. The resulting slurry was added to the Qiagen QIAamp DNA Microbiome Kit (Qiagen, Germantown, MD, USA), and DNA was extracted according to the manufacturer’s protocol. Four reagent blanks were generated alongside experimental samples, without coral biomass. The extracted DNA was then submitted to the University of Florida’s Interdisciplinary Center for Biotechnology Research NextGen DNA Sequencing core (RRID:SCR_019152) for library preparation and sequencing on an Illumina NovaSeq 6000 (Illumina, San Diego, CA, USA) in 150-bp paired-end format. Reagent blanks were sequenced on an Illumina MiSeq. Further details are in the SI Methods.

### Bioinformatics

An overview of the bioinformatics pipeline we employed to target bacteria and archaea within the coral holobiont is outlined in Fig. S1. Detailed parameters can be found in the SI Methods. We assessed the quality of raw reads using FastQC v.0.11.7, then trimmed and filtered the raw reads using trimmomatic v.0.39 [44]. To remove host and endosymbiont DNA and begin selecting for prokaryotic genomic content, we used Bowtie2 v.2.4.2 [45] to map trimmed reads against the following genomes: *O. faveolata*, *Breviolum minutum*, *Symbiodinium* sp. Clade C, and *Durusdinium sp.* We assembled the remaining reads that did not map to the eukaryotic genomes (host/endosymbiont-cleaned reads) from each of the 41 corals individually using MegaHit v.1.1.3 [46] and assessed the assemblies with QUAST v.5.2.0 [47] (Table S2). To evaluate the broad prokaryotic versus eukaryotic content of the assemblies, we used EukRep v.0.6.7 [48] followed by QUAST v.5.2.0.

We further selected the prokaryotic component of the host/endosymbiont-cleaned reads (Table S2) by mapping them to the eukaryotic contigs identified by EukRep and removing eukaryotic reads with Bowtie2 v.2.4.2 [45]. We then generated a co-assembly of all 41 coral samples with MegaHit v.1.1.3 [46], then evaluated it with QUAST v.5.2.0. We used EukRep v.0.6.7 again to keep the prokaryotic portion of the co-assembly for annotation and analysis.

To predict protein-coding genes, we used Prodigal v.2.6.3 [49]. We taxonomically annotated the predicted genes (amino acid fasta) with MMseqs2 v.2/14, employing the GTDB database v.214.1 [50]. For functional annotations, we used GhostKOALA [51]. We quantified the abundance of predicted genes from the prokaryote co-assembly using Salmon v.1.10.1 [52] to generate a table of read counts based on the length and relative abundance of the gene in the prokaryote co-assembly that was used for statistical analyses.

We used the resistance gene identifier (RGI) v.6.0.2 to find antibiotic resistance genes (ARGs) in the predicted genes from the prokaryote co-assembly (amino acid fasta) [53]. To identify biosynthetic gene clusters (BGCs), we used antiSMASH v.7.0.0 on the prokaryotic co-assembly generated by EukRep. We manually curated the antiSMASH and MIBiG results to identify antimicrobial BGCs (Table S3). CoverM v.0.6.1 was used to measure mean coverage of BGCs in the eukaryote-removed reads [54] that was converted to presence/absence for statistical analysis. Further details and parameters for the bioinformatics analysis are in the SI Methods.

### Statistical analyses

For statistical tests, we used the read counts generated by Salmon for each coral sample of the predicted genes in the co-assembly (247 254 genes). We analyzed the beta diversity of functional genes using Aitchison distance to account for compositional data [55]. To test how colony-specific covariates (coral genotype, fate, disease condition at sampling, Symbiodiniaceae composition, and reef location) explained variability in the functional microbiome, we used the adonis2 function [56] in vegan v.2.6.6.1. For all tests, genotypic clones were treated as separate individuals (Fig. S2a, SI Results, Table S4). To investigate dispersion of the functional genes, we used the betadisper function in vegan v.2.6.6.1. Then, we conducted a Kruskal–Wallis or ANOVA test followed by either a Wilcoxon or Tukey *post-hoc* test to generate adjusted *P*-values.

To identify genes that may be involved in disease resistance, we conducted a differential abundance test in corncob between unaffected corals (baseline) and the other colony fates. We used corncob v.0.4.1 [57] on counts of the 31 695 functionally annotated genes and controlled for the composition of Symbiodiniaceae and for differential variance. Significant differences were evaluated using the parametric Wald test, with a Benjamini–Hochberg false discovery rate correction of 0.05. Prior to plotting, Kyoto Encyclopedia of Genes and Genomes (KEGG) annotations were retrieved for each KO ID using the KEGG API in R v4.3.2 [58].

To test whether repeated amoxicillin treatments impacted the richness and diversity of beta-lactamase ARGs, we used the package breakaway v4.8.4 [59] to estimate the richness of beta-lactamase genes. We tested for the effect of amoxicillin treatments and recent treatment history on richness using the betta function [60]. We additionally tested for the effect of these variables on the beta diversity of beta-lactamase genes using the methods described above. To test whether BGCs were differentially prevalent across colony fates, we used a Fisher’s exact test incorporating a Monte-Carlo simulation, followed by a Benjamini–Hochberg multiple test correction in R v4.3.2. Further statistical details are in the SI Methods.

## Results

### Disease history and characteristics of *O. faveolata* colonies

*Orbicella faveolata* colonies from 2 reefs in the lower Florida Keys were monitored for up to 27 months prior to sampling in June 2021 for SCTLD and treated in cases of active lesions (Fig. 1d, Table S1). Total topical amoxicillin treatments applied to colonies before sampling ranged from 0 (on colonies that had never been observed with SCTLD) to 779 (Table S1). The colonies with the most treatments were generally large (up to 32 m^2^ of estimated live tissue area) and often had new lesions during each visit (Fig. 1b; Tables S1).

After 4 to 5 years of bimonthly observations, all colonies survived, and 20% of the selected colonies were never observed with SCTLD (unaffected) (Fig. 2). In contrast, 49% exhibited SCTLD lesions before, during, and after sampling (diseased), and 10% of colonies were diseased prior to sampling but resisted infection for the entire post-sampling period (recovered). In addition, 22% of colonies were unaffected during sampling, but developed lesions after sampling (will be diseased) and included both previously affected and previously unaffected colonies. During tissue sampling, 49% exhibited active SCTLD lesions and 51% were apparently healthy.

**Figure 2:**
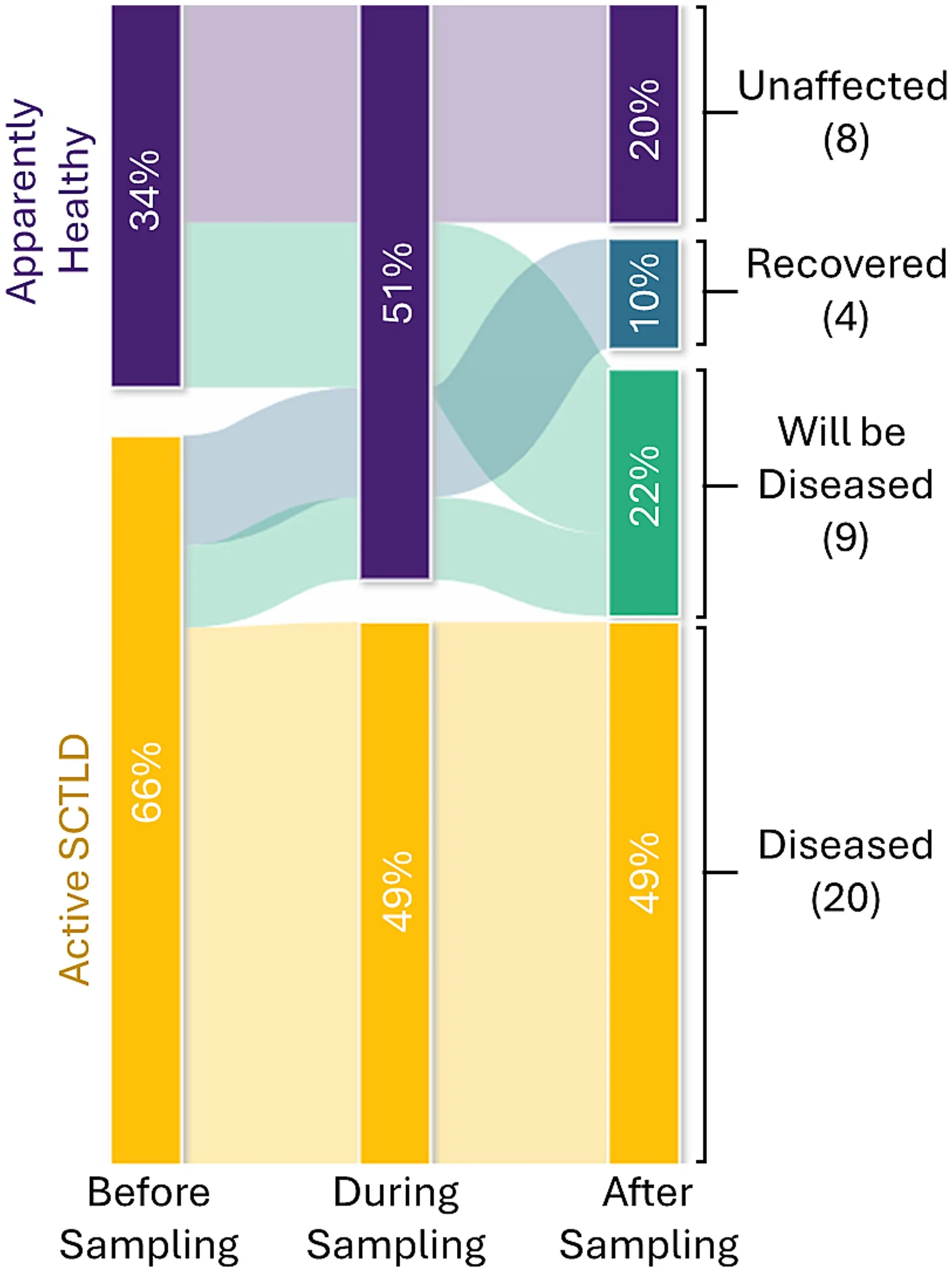
Colony fate categories were based on fate-tracking of 41 *O. faveolata* colonies every 2 months for up to 5 years for the presence of stony coral tissue loss disease. Four “colony fate” categories (number of colonies in parentheses) were defined based on the presence of SCTLD at any point before sampling (as early as 2019), during sampling (June 12–13, 2021), or after sampling (July 2021–July 2024). These categories were: unaffected (never diseased), recovered (diseased before sampling, but not during or after), will be diseased (not diseased during sampling, but diseased afterwards), and diseased (diseased before, during, and after sampling). Colonies with active SCTLD were treated throughout the study. Prior to microbiome sampling, all apparently healthy colonies in the first bar were not treated (34%, *n* = 14), while those with active SCTLD were treated (66%, *n* = 27). Colonies in the will be diseased category include 6 that were apparently healthy and received no antibiotic treatments prior to microbiome sampling and 3 with SCTLD and antibiotic treatments at least 12 months prior to microbiome sampling.

We quantified Symbiodiniaceae community composition via qPCR, and for all but one coral sample, *Breviolum* was the main genus of Symbiodiniaceae present (Fig. S3), and 46.3% of the colonies harbored small proportions of *Cladocopium, Durusdinium,* or both genera in addition to *Breviolum.* One coral sample contained both *Cladocopium* and *Durusdinium*, but no *Breviolum*.

### *In silico* selection of the coral prokaryotic microbiome

Sequencing of metagenomes from apparently healthy coral tissues resulted in approximately 132 million reads per sample that were filtered to approximately 30.4 million reads per coral sample after selecting for prokaryotic genomic content (Table S2). We additionally generated 16S rRNA analyses of four DNA extraction reagent blanks (SI Results). We removed on average 74.35% of reads deemed eukaryotic from the metagenome reads (see Materials and methods, Table S2). The prokaryotic portion of the co-assembly was 11% of the total length of the assembly, was 287 437 943 bp long, had an N50 of 1903 bp, and a longest contig of 367 269 bp. Following protein-coding gene prediction, the prokaryotic co-assembly contained 247 254 genes that were used for downstream analyses. The prokaryotic co-assembly was taxonomically classified to 172 different bacterial and archaeal classes, and an average of 79.7% of sequences were unclassified at the domain level (Fig. S4).

### Markers of disease resilience in the prokaryotic functional microbiome

Symbiodiniaceae community composition (qPCR-based), colony fate, and colony condition at sampling explained 53.4% of the variation in prokaryotic functional microbiome beta diversity (PERMANOVA, 999 permutations, *P* < .05, Fig. 3, Table S4). Symbiodiniaceae community composition and colony fate explained 32.8% and 12.7% of the variance, respectively (Fig. 3, Table S4). Condition of the colony at sampling (apparently healthy or actively diseased) explained 7.9% of the variance (Fig. 3, Table S4). Reef and coral host genotype did not explain prokaryotic functional microbiome composition (PERMANOVA *P* > .05, Fig. S2).

**Figure 3:**
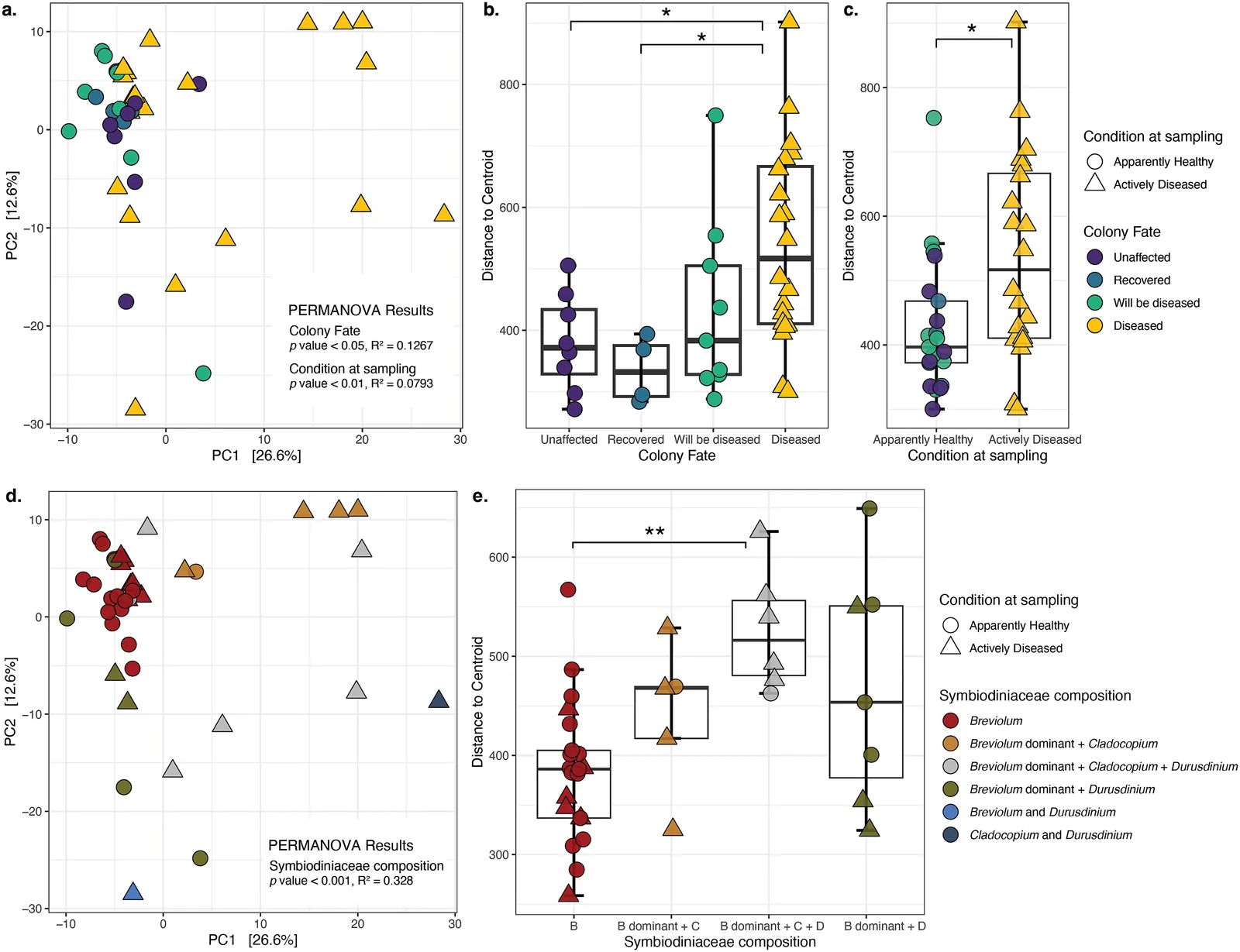
Functional microbiome composition in *O. faveolata* is explained by colony fate, coral condition at sampling, and Symbiodiniaceae composition (PERMANOVA, 999 permutations, *P* < .05). (a) Principal components analysis (PCA) of coral tissue prokaryotic functional microbiome beta diversity (Aitchison distance) colored by colony fate with point shape reflecting the condition of the coral at sampling. (b) Prokaryotic functional beta diversity dispersion measured as the distance to centroid was greater in colonies with diseased fates relative to recovered or unaffected fates (Wilcoxon rank sum test, *P < .05). (c) Prokaryotic functional beta diversity dispersion measured as the distance to centroid was greater in actively diseased colonies relative to apparently healthy colonies (Wilcoxon rank sum test, *P < .05). (d) PCA as in (a) colored by Symbiodiniaceae composition. (e) Functional prokaryotic beta dispersion differed across Symbiodiniaceae composition groups, with corals containing *Breviolum* dominant + *Cladocopium* + *Durusdinium* exhibiting greater dispersion than *Breviolum-*only tissues (Tukey Honest Significant Differences *post-hoc* test, **P < .01). Note that the *Cladocopium* and *Durusdinium* and the *Breviolum* and *Durusdinium* groupings are omitted from panel (d) because there was only one coral in each group. All points are colored by Symbiodiniaceae composition groupings, and shape reflects the colony condition at the time of sampling. The groupings *Breviolum* and *Durusdinium,* and *Cladocopium* and *Durusdinium* contain both genera making up at least 10% of symbiont community. See Fig. S3 for a visualization of the photoendosymbiont composition of all corals.

Dispersion in the prokaryotic functional microbiome was increased in actively diseased colonies relative to apparently healthy colonies (Wilcoxon rank sum test *P* < .05, Fig. 3c). Functional microbiomes from diseased coral fates were more dispersed compared to those of recovered and unaffected fates, but not to those of the will be diseased fate (Wilcoxon Rank Sum *post-hoc* test, adjusted *P* < .05, Fig. 3b). For corals in the will be diseased fate, the microbial dispersion was not related to months until disease onset, to presence or absence of disease lesions prior to sampling, nor to history of antibiotic treatment (linear model or Wilcoxon rank sum test, *P* > .05, Fig. S5). Increased functional microbiome variability also coincided with differences in Symbiodiniaceae composition (ANOVA *P* < .001, Fig. 3e). Compared to colonies with exclusively *Breviolum* symbionts, those with small proportions of both *Cladocopium* and *Durusdinium* harbored more dispersed prokaryotic functional microbiomes (Tukey HSD *post-hoc* test adjusted *P* < .05, Fig. 3e).

We identified 392 functional genes that were differentially abundant in the recovered, will be diseased, and diseased coral fates relative to corals unaffected for the duration of our monitoring period (Corncob adjusted *P* < .05, Table S5). Colonies that recovered from a prior SCTLD infection and remained disease-free harbored greater abundances of genes from the Actinomycetota and Bacillota phyla for vitamin and cofactor metabolism, quorum sensing, beta-lactam resistance, membrane transport, and secretion systems relative to unaffected colonies (Fig. 4, Table S5). In contrast, unaffected tissue of actively diseased colonies had lower abundances of genes relative to unaffected colonies, including those for vitamin metabolism, biosynthesis of antibiotics (phenazine, novobiocin, carbapenem, monobactam), membrane transport genes, quorum sensing, and beta-lactam resistance genes (Fig. 4, Table S5). Despite the colonies being apparently healthy at sampling, corals with will be diseased fates had microbiomes with lower abundances of most cofactor and vitamin metabolism genes, secondary metabolite biosynthesis genes, and transport and signaling genes relative to unaffected colonies, which mirrored the patterns of diseased colonies (Fig. 4, Table S5).

**Figure 4:**
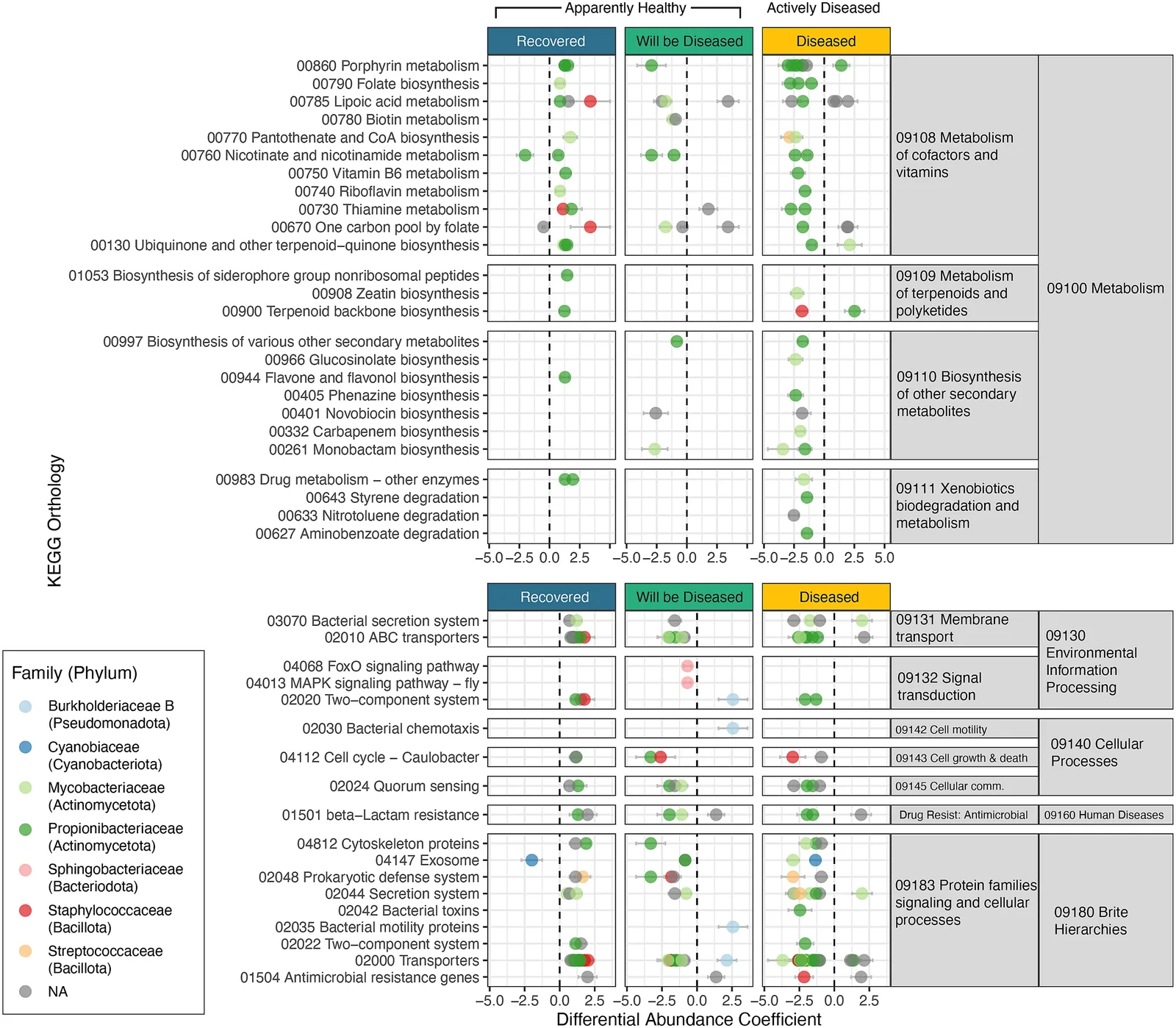
Genes involved in metabolism of secondary metabolites and other natural products, as well as those involved in signal transduction and cellular processes, were less abundant in diseased and corals that will be diseased categories as compared to unaffected colonies, but enriched in corals recovered from SCTLD (Corncob, Benjamini–Hochberg false discovery rate corrected *P* < .05). The differential abundance coefficient represents how much the modeled relative abundance of a gene was enriched or depleted relative to the baseline (unaffected colonies, dotted line at zero). Error bars reflect 95% confidence intervals around the coefficient. Results are organized by KEGG hierarchy, with the top panel representing metabolism genes, and the bottom panel representing all other processes. There are some points (genes) that are repeated due to their involvement in multiple pathways and roles. The color represents the family and phylum-level annotations for a given predicted gene from the GTDB database. These differentially abundant genes are a subset of all. See Table S6 for a list of all 392 genes and the comparisons for which they were significantly differentially abundant.

### Coral microbiomes encode diverse genes for antimicrobial resistance and biosynthesis

The 14 colonies that were never-diseased prior to sampling were not treated with amoxicillin, while the other 27 with previous SCTLD lesions were treated at some point (and sometimes repeatedly) before sampling (Table S1). Despite the potential selective pressure exerted by repeated application of up to hundreds of treatments applied in the months to years prior to metagenomic sampling, we did not find a correlation between the number of amoxicillin treatments (raw and scaled to m^2^ of live coral tissue) or treatment history (categorical: never treated, treatment >2 months prior, recent treatment <2 months prior) and the richness of beta-lactamase genes within the coral microbiome (betta test *P* > .05, Fig. 5a). Beta diversity of beta-lactamase genes also did not differ due to the number of treatments or treatment history (PERMANOVA *P* > .05, Fig. 5b). Of the 53 467 beta-lactamase genes present in the coral microbiome, most were taxonomically unclassified, but some were classified as *Actinomycetia*, *Alphaproteobacteria*, *Clostridia*, or *Gammaproteobacteria* (Fig. 5c, Table S6).

**Figure 5:**
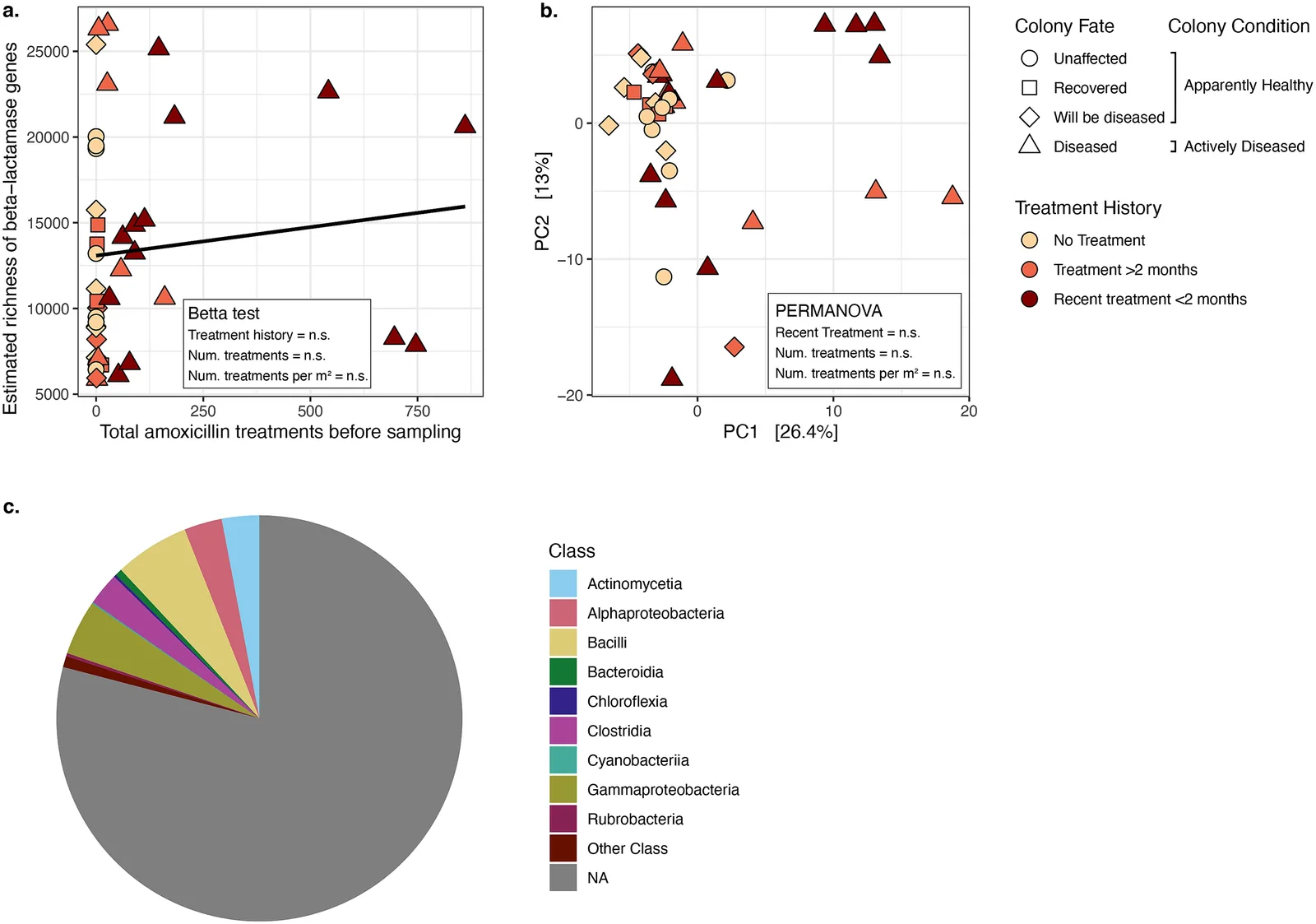
Diverse beta-lactamase genes exist within the coral microbiome regardless of application of the antibiotic amoxicillin. (a) The estimated richness (breakaway) of the beta-lactamase genes in each sample did not significantly increase with amoxicillin treatments or recent amoxicillin application [betta test *P* > .05 for total number of amoxicillin treatments (continuous), total number of amoxicillin treatments scaled per m^2^ of live tissue area (continuous), and treatment history (categorical)]. The line represents a linear regression. n.s. = not significant from the betta test for heterogeneity of total diversity. (b) PCA showing the beta diversity of beta-lactamase genes in the coral microbiome did not differ due to the number of treatments (raw and scaled to tissue area) or recent treatment application (PERMANOVA, *P* > .05). Points in (a) and (b) are shaped based on the colony fate and colored based on treatment history. (c) Taxonomic affiliation of the 53 467 individual beta-lactamase genes depicted as a pie chart indicated most were unclassified (NA), and a small proportion were annotated at the class taxonomic level. The top nine most abundant classes are shown, and the “Other Class” includes the remaining 94 classes.

We investigated the diverse BGCs within the coral microbiome and identified 102 BGCs, of which 56 encoded putatively probiotic antimicrobial compounds (Fig. 6, Table S3, Fig. S6). The 56 BGCs encoding antimicrobial compounds were prevalent across coral colonies, regardless of their colony fate (Fisher’s exact test, Benjamini–Hochberg adjusted *P* > .05, Fig. 6). They included BGCs that matched biosynthetic genes for a variety of marine-derived compounds, such as curacin A, bryostatin, and polytheonamide A and B (Fig. 6). Some of the most highly prevalent BGCs had matches to xenematide, toyoncin, ergovaline, carnobacteriocin XY, strobilurin A, and polytheonamide A and B (Fig. 6). Overall, the closest matches in the MIBiG database to the coral microbiome BGCs identified in this study encoded 65 different compounds, of which 43 were antimicrobial or toxins (Table S3, Fig. S6).

**Figure 6:**
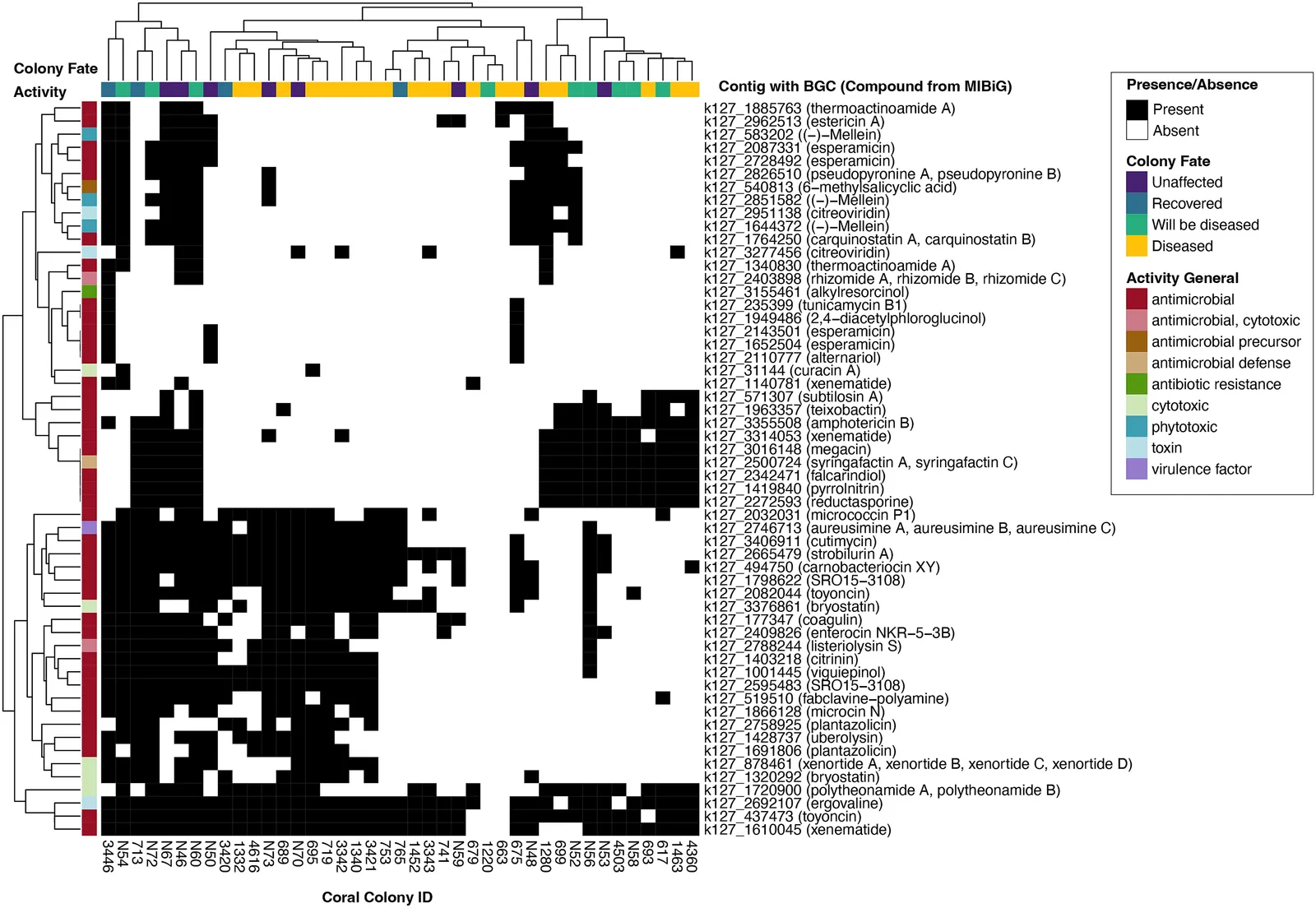
The coral microbiome encodes diverse potential for antimicrobial activity regardless of disease condition (Fisher’s exact test, Benjamini–Hochberg false discovery rate corrected *P* > .05). Heatmap displays presence or absence of a contig containing the potential antimicrobial-encoding biosynthetic gene cluster (BGC) across all coral colonies and is clustered by Euclidean distance. Presence was defined as a nonzero mean coverage of at least 10% of the contig in the eukaryote-removed reads, and the compound label reflects the compound encoded by the closest MIBiG database match returned by antiSMASH (see Materials and methods). Coral colonies are colored to reflect the fate of the colony, and contigs are colored to reflect the inferred activity of the BGC.

## Discussion

We undertook 4 to 5 years of bimonthly fate-tracking and antibiotic interventions on *O. faveolata* colonies and identified intraspecific variation in resistance to SCTLD. Following deep sequencing of the prokaryotic functional microbiome, we found evidence that vitamin biosynthesis and putative probiotic genes are associated with resilience in treated and untreated colonies, and may improve host health in the face of disease exposure. Conversely, dysbiotic functional microbiomes were a marker of disease. In these large *O. faveolata* colonies, the microbiome harbored diverse beta-lactamase genes, even in colonies that were never treated with amoxicillin. As SCTLD continues to impact reefs with no known etiological agent or fully effective cure, these data provide insight into the microbial markers that may underlie the variable susceptibility of *O. faveolata* colonies to SCTLD.

Apparently healthy tissue of *O. faveolata* impacted by SCTLD had more variable prokaryotic functional microbiomes than unaffected individuals, which may be an indicator of systemic dysbiosis. The Anna Karenina hypothesis offers a useful analogy for dysbiosis in diseased animal microbiomes: “All happy families are alike; each unhappy family is unhappy in its own way” [61]. In our study, all recovered and unaffected colonies harbored low functional microbiome variability, while diseased colonies were more dispersed, reflecting dysbiosis. Interestingly, will be diseased colonies, which were apparently healthy during sampling but became diseased following sampling, had intermediate microbial functional variability. Although the increased variability within the will be diseased colonies did not relate to time until infection or to past infection history, this overall stochasticity of prokaryotic functional potential within healthy corals prone to infection may be a marker of SCTLD. Previous prokaryotic taxonomic investigations found increased dispersion associated with healthy tissue in SCTLD-affected colonies [20, 23], and here we identify microbial functional dispersion in corals not yet infected with SCTLD. Therefore, SCTLD may first present as a functional dysbiosis, potentially triggered by bacteriophages, that leads to disease [31]. SCTLD-susceptible corals are known to be dysbiotic over time [62], and while we did not sample multiple timepoints for metagenomics here, we hypothesize that those disease-susceptible *O. faveolata* colonies would be most variable in their functional microbiome. Contrasting this, in previous coral bleaching work, lower microbial functional dispersion was associated with increased putative probiotic potential to support coral health following a stressor [63]. Here, both colonies that recovered from disease and those unaffected by disease had the lowest dispersion. In contrast to a dysbiosis signal, our work suggests a consistent suite of microbial functions represents the microbiome that supports host resilience and resistance to disease.

Functional stochasticity in the coral microbiome also coincided with varied Symbiodiniaceae composition. Corals with only *Breviolum* symbionts generally had less microbial dispersion than coral colonies that also included small amounts of *Cladocopium* and *Durusdinium*. Connection between Symbiodiniaceae, a large component of the coral holobiont, with the prokaryotic microbiome has been reported previously [64, 65]. Here, the composition of Symbiodiniaceae explained 33% of prokaryotic functional variability, linking algal symbionts to the prokaryotic microbiome. The Symbiodiniaceae-associated microbiome is known to influence cnidarian host fitness [66]. Given that the functional microbiome is associated with both fate and Symbiodiniaceae, it may be that inter-kingdom interactions in the coral holobiont influence host SCTLD resilience.

Microorganisms within resilient, or recovered, corals harbored increased potential to support host health compared to corals that ultimately were affected by disease. Recovered coral functional microbiomes had higher relative abundances of B vitamin metabolism genes compared to all other groups of corals. Corals and Symbiodiniaceae symbionts are unable to produce B vitamins, and bacteria produce B vitamins to support the coral and symbiont host metabolism [14, 67, 68]. Additionally, quorum sensing was a marker of resilient corals. While quorum sensing is associated with coral disease and bleaching [69, 70], some of the differentially abundant quorum-sensing genes here included components of DAHP synthase and anthranilate synthase, important in tryptophan amino acid biosynthesis [71]. Tryptophan is exuded by corals [72], and nonpathogenic coral-associated bacteria exhibit chemotaxis toward it [73]. Our results suggest quorum-sensing capabilities may be important for maintaining natural coral-microbial symbioses and increasing host resilience to disease. In contrast to recovered corals, actively diseased and apparently healthy corals that contracted disease after sampling had functional microbiomes with lower abundances of vitamin B metabolism and quorum-sensing genes compared to unaffected colonies, which may have compromised host and symbiont fitness. These were predominantly genes from members of the Actinomycetota, a group commonly associated with coral health [74]. Additionally, compromised coral microbiomes had lower abundances of secondary metabolite and xenobiotics biosynthesis pathways. These pathways can be important for defense against infection [75], and lowered potential for these may have additionally predisposed these colonies to the disease they ultimately contracted.

Amoxicillin treatments are the primary intervention strategy for halting SCTLD lesions in the Caribbean [8, 39, 76–79]. Although effective at preventing SCTLD-related mortality, these treatments have raised concerns over the potential development of antibiotic resistance [80, 81]. There was no evidence in our study that treatments selected for an increased richness or diversity of beta-lactamase genes, even with corals that received hundreds of individual lesion treatments in the years prior to sampling. Our findings were complicated by the variability in treatment history of each coral. One treatment was defined as the application of amoxicillin paste onto an active lesion, regardless of lesion size, location on the colony relative to the sample location, or colony size. For example, some of the largest colonies received hundreds of treatments that were often meters from the sampling location. Additionally, these applications occurred 1 month to over 2 years prior to sampling. We accounted for these complications by scaling treatments to colony size and testing if treatment history impacted beta-lactamase diversity. Our findings offer an optimistic view that repeated topical antibiotic treatments did not select for greater potential for antibiotic resistance within the coral microbiome. Additionally, recent findings indicate these localized, topical treatments do not affect coral physiology [82], reproduction [83, 84], or gene expression [85], and treatments do not appear to increase the expression of ARGs [86]. Indeed, we identified about 200 000 ARGs, representing the majority of the 247 254 protein-coding prokaryotic genes in the co-assembly, a high proportion of our genes that may be in part due to our inclusion of “loose” hits to detect potentially novel marine ARGs [87]. Similarly, ARGs were prevalent in untreated, SCTLD-impacted coral tissue [30]. Our research indicates that antibiotic resistance may not be a signal of disease treatments, but rather a native feature of the coral microbiome [86]. While encouraging, antibiotics are known to select for antibiotic resistance, and moving forward, managers and scientists aiming to curb SCTLD should continue to be prudent with environmental applications of amoxicillin.

Antimicrobial and bioactive biosynthetic gene clusters were prevalent across coral microbiomes, regardless of colony fate, and may play an important role in host defense. More than half of the BGCs we identified in the prokaryotic functional microbiome encoded potential antimicrobial or toxin production genes. The detection of broad and diverse pathways for antimicrobial production may not be surprising given the known ability of coral-associated bacteria to synthesize antimicrobial compounds that inhibit potential pathogens [88–90]. For example, *Pseudoalteromonas* bacterial isolates from healthy *O. faveolata* in SCTLD-impacted areas are known to produce the antibiotic alteramide A [91], and one *Pseudoalteromonas* strain has been an effective probiotic treatment for SCTLD [15, 92]. While we did not detect genes for the synthesis of alteramide A, our *in silico* approaches identified multiple other antibiotic and bioactive production pathways. Only three matched natural products isolated and described from marine environments, highlighting the paucity of marine research compared to those in terrestrial, agricultural, or human disease-related environments. These compounds: curacin A, bryostatin, and polytheonamide A and B, all play a role in host defense and have demonstrated anti-cancer activity. Curacin A was isolated from the marine cyanobacterium, *Lyngbya majuscula*, from Caribbean reef habitats and kills both human cell lines and brine shrimp [93]. We found genes encoding production of this compound present in one apparently healthy and one diseased *O. faveolata.* Bryostatins may provide a protective role for host larvae by deterring predation [94]. Like curacin A, bryostatin has also demonstrated anticancer and cytotoxic activity [95]. Two bryostatin biosynthetic gene clusters identified in both actively diseased and apparently healthy *O. faveolata* microbiomes match (0.11–0.16 similarity score) those from the uncultured symbiont of a marine bryozoan, *Candidatus* Endobugula sertula [96]. Polytheonamide A and B were isolated from a Pacific reef sponge, *Theonella swinhoei*, and show cytotoxic activity against cancer cells [97]. These products are likely synthesized by uncultivated *Candidatus* Entotheonella bacterial symbionts [98], which are known to harbor immense bioactive potential, and may serve as chemical defense for the sessile host sponge [99]. Like bryostatin and curacin A, genes for polytheonamides A and B were present in metagenomes of both apparently healthy and actively diseased colonies. Considering we sampled apparently healthy tissue on all colonies, perhaps the presence of genes encoding production of cytotoxic compounds is a reflection of the native and significant potential for coral microbiomes to harbor bacteria that protect the coral host.

Some of the most prevalent biosynthetic gene clusters were those that may play a role in host defense and homeostasis. Xenematide, toyoncin, and ergovaline production genes were present in over 90% of the corals, and carnobacteriocin XY and strobilurin A were present in 68% of the sampled corals. Xenematide is produced by bacterial symbionts of nematodes to maintain symbiosis within the nematode host [100]. Toyoncin is a bacteriocin isolated from a *Bacillus cereus* strain that is antimicrobial against foodborne pathogens [101]. While mainly considered a food contaminant, *B. cereus* has also been isolated from marine sponges, where it also produces antibiotics [102]. Ergovaline and strobilurin A are both fungal-based antimicrobial compounds [103, 104], though the nearest BLAST-based matches to the core biosynthetic gene from antiSMASH were bacterial, suggesting similar antimicrobial compounds across domains or potential horizontal gene transfer events. Carnobacteriocin XY was isolated from lactic acid bacteria, which are common in food, though *Carnobacterium* species that produce antimicrobial compounds are also found in marine habitats [105]. The high prevalence of these antimicrobial and defensive compounds is likely reflective of the *O. faveolata* microbiome’s ability to support host homeostasis and prevent predation or infection, which would be essential within apparently healthy tissue, regardless of infection status of the coral host overall.

While some *O. faveolata* colonies exhibited repeated SCTLD infections, over 4 to 5 years of *in situ* monitoring, 20% of our selected corals resisted infection, and 10% were resilient and recovered from SCTLD. By combining this extensive disease history with the characterization of the functional potential of bacterial microbiomes, we provide evidence that this resilience to disease may be due to the ability of the coral microbiome to synthesize antimicrobial products that protect against invading pathogens within the apparently healthy tissue. The survival of these large coral colonies was also due to antibiotic interventions that halted disease progression, yet these treatments did not alter the natively diverse suite of antibiotic resistance genes present within the coral microbiome. In addition to identifying markers of resilience, we uncovered high microbial dispersion, or dysbiosis, present in unaffected colonies that may have predisposed them to the disease they contracted. While causative agents of this widespread disease remain elusive, our efforts uncovered a suite of putative probiotic markers of resilience to SCTLD within the coral microbiome. This supports the growing emphasis on developing probiotic treatments and microbial consortia to improve coral host fitness in the face of disease and other widespread stressors [15, 106].

## Supplementary Material

ISMECOMMUN-D-26-00011_Supplementary_Figures_ycag278

Table_S1_Coral_treatment_history_and_monitoring_ycag278

Table_S2_Reads_through_bioinf_pipeline_ycag278

Table_S3_Biosynthetic_Gene_Clusters_ycag278

Table_S4_PERMANOVA_Results_ycag278

Table_S5_Differential_Abundance_Results_Colony_Fate_ycag278

Table_S6_Antibiotic_Resistance_Genes_ycag278

Table_S7_Blank_Sample_16s_rRNA_ASVs_and_taxonomy_ycag278

ISMECOMMUN-D-26-00011_Supplementary_Information_ycag278

## Data Availability

The raw sequence reads are publicly available in the NCBI Sequence Read Archive under BioProject accession number PRJNA925892. The individual coral metagenome assemblies, the co-assembly, predicted proteins, annotations, and abundances are publicly available via Zenodo dataset https://doi.org/10.5281/zenodo.11493775 version 2.0. Scripts containing the code with parameters for bioinformatics as well as the code to reproduce the analyses and figures within this manuscript are available on GitHub at https://github.com/meyermicrobiolab/FLK_SCTLD_Resistance_OFAV/.

## Notes

Present address: Cynthia C. Becker, Biology Department, Ithaca College, Ithaca, NY 14850, United States.
