# Supplementary material for: Markers of resilience to stony coral tissue loss disease and putative probiotic functions in the microbiomes of the coral, Orbicella faveolata
Source: ISME Commun. 2026 Oct 1;6(1):ycag278. doi: 10.1093/ismeco/ycag278 (PMC13685810; doi:10.1093/ismeco/ycag278)
Supplement: Supplementary file 2 [file ismecommun-d-26-00011_supplementary_information_ycag278.docx]

# Supplementary Information for “Markers of resilience to stony coral tissue loss disease and putative probiotic functions in the microbiomes of the coral, *Orbicella faveolata*”

Cynthia C. Becker^a,*^, Allison R. Cauvin^b^, Karen L. Neely^c^, Caroline Dennison^d^, Andrew C. Baker^d^, Brian K. Walker^c^, Julie L. Meyer^a,#^

^a^Department of Soil, Water, and Ecosystem Sciences, Genetics Institute, Institute of Food and Agricultural Sciences, University of Florida, Gainesville, Florida, USA

^b^School of Natural Resources and Environment, University of Florida, Gainesville, Florida, USA

^c^National Coral Reef Institute, Nova Southeastern University, Ft. Lauderdale, Florida, USA
^d^Department of Marine Biology and Ecology, Rosenstiel School of Marine, Atmospheric, and Earth Science, University of Miami, Miami, Florida, USA

^#^Corresponding Author: Julie L. Meyer, [juliemeyer@ufl.edu](mailto:juliemeyer@ufl.edu),

Genetics Institute, Rm 304, Cancer and Genetics Research Complex

2033 Mowry Rd. University of Florida, Gainesville, Florida 32611

*Present address: Biology Department, Ithaca College, Ithaca, NY

# Methods

### Study area and collection

Core samples were taken from 41 *Orbicella faveolata* colonies at two reef sites in the lower Florida Keys: Looe Key and Sand Key (Figure 1a). Both sites are spur-and-groove forereefs, and the bases of all colonies were at 3 to 9 meters depth. The two sites are approximately 49 km apart, and within each site, sampled corals were all within 0.14 km (Looe Key) to 0.3 km (Sand Key) of each other.

The selected corals were part of a long-term SCTLD intervention and monitoring project across multiple species and sites [[1–3]](https://paperpile.com/c/wkf7pu/otWIW+K9gHF+RrBv3). Samples from the selected corals were collected by the SCTLD Resistance Research Consortium (RRC), which included the measurement of over a dozen holobiont traits [[4]](https://paperpile.com/c/wkf7pu/YCJ0L) SCTLD was first documented at Looe Key in April 2018, and at Sand Key in early-mid 2019. In-water amoxicillin interventions and fate-tracking were initiated at Looe Key in March 2019 and at Sand Key in October 2019. Each site was visited approximately every two months, and any coral affected by SCTLD was tagged with a numbered cattle tag, mapped and photographed for revisitation, and treated on active disease lesions with a topical amoxicillin paste. The paste was an 8:1 by weight mixture of Ocean Alchemist’s Base2b (CoralCure) mixed with 98% amoxicillin trihydrate powder no more than 24 hours before application and placed into 60cc catheter syringes which were taken underwater. The paste was applied by hand in an approximately 1 cm wide line across any active lesions and pressed into the skeleton and coral tissue. Prior to June 2021, these efforts included 73 *O. faveolata* colonies at Sand Key and 547 *O. faveolata* colonies at Looe Key. From these candidates, we selected 11 colonies from Sand Key and 16 from Looe Key for sampling. Colonies were selected to be geographically near each other and to represent individuals that exhibited frequent reinfections (active lesions during most semi-monthly visits) and those who had only had active SCTLD lesions once. Additionally, we selected 14 “resistant” colonies (6 at Sand Key, 8 at Looe Key) that did not appear to have ever had SCTLD. Though we had not specifically fate-tracked these corals before selection, we chose colonies that had nearly 100% coral cover and were within the regularly monitored areas (we swam over them every two months since the start of monitoring), and can thus assume they had not had any SCTLD lesions in the months to years prior to selection and sampling. After sampling, all corals (including those tagged as resistant) were revisited approximately every two months until July 2024 for monitoring and treatments if necessary. During each visit (before and after sampling) the health status of the coral as well as the number of treatments (if applied) was recorded. A treatment was defined as the application of amoxicillin paste to an active lesion along the live tissue border. As such, if a single lesion affected multiple tissue isolates, that lesion could receive multiple treatments. Treatments could range in size from ~1 cm in length to >100 cm in length. Treatments were applied as needed throughout the entire colony, and were summed across all previous monitoring events (from 2019 onwards).

Cores from the 41 colonies were collected on June 12-13, 2021. Divers on SCUBA used a clean, separate 12 mm leather punch to extract a 1 cm core from apparently healthy tissue on each colony. Separate cores were collected in similar locations on the colony for metagenomics and for symbiont analyses. Core sites on each colony were selected to be distant from any active SCTLD lesions or other sources of recent mortality (Figure 1C). Each core was placed into a labeled bag and immediately transported to the surface for processing while the coring team moved to the next colony (<10 minutes from coring to processing). At the surface, tissue samples were preserved in DNA/RNA Shield (Zymo Research, Irvine, CA, USA) and kept on ice during transportation back to the lab. All samples were subsequently stored at −80ºC until processing.

### Colony characteristics

The initial selection of *O. faveolata* colonies was based on “fully resistant” (no past or present disease lesions), “high resistance” (had disease lesions once, but after treatment never developed new lesions), and “low resistance” (repeatedly developed disease lesions). However, in the three years of fate-tracked monitoring that followed sampling, some corals shifted from one category into another, and so we developed new categories of colony fate that incorporated the full health histories of the colonies. Each colony was classed as either: 1) “diseased” (had SCTLD before sampling, during sampling, and after sampling), 2) “will be diseased” (was healthy at the time of sampling, but developed SCTLD lesions at some point (from 4 to 30 months) afterwards), 3) “recovered” (coral had SCTLD before sampling, but not during sampling and at no point after sampling), or 4) “unaffected” (coral was never observed with SCTLD).

The coral host genotype was identified from adjacent samples analyzed by and reported in Klein et al [[5]](https://paperpile.com/c/wkf7pu/XRjV8). Algal symbiont composition was determined using an actin-based quantitative PCR (qPCR) assay as previously described [[6]](https://paperpile.com/c/wkf7pu/5BfNg). The qPCR assay enabled quantitation of the three different predominant genera within the corals: *Breviolum, Durusdinium, and Cladocopium.* Based on the prevalence, we determined if the genus was dominant (>90% of the community) or a background member of the symbiont community (<10% of the community). We used the relative proportions to generate five categories that reflected the Symbiodiniaceae community composition, which are depicted in Figure S3.

### Coral microbiome approach background

## Although the coral holobiont was one of the earliest systems for shotgun metagenomic sequencing [[7, 8]](https://paperpile.com/c/wkf7pu/0kmSF+dLQ3l), coral metagenomic studies that target bacterial functional genes are limited due to technical challenges. There is a stark difference in genome size between the host and symbiotic algae compared to prokaryotes. The *O. faveolata* genome is approximately 470 - 536 Mb while algal symbionts in the genus *Breviolum*, the dominant algal genera in this study, have genomes of approximately 616 Mb [[9–11]](https://paperpile.com/c/wkf7pu/L8Pid+3W5F9+NhVcf). Other Symbiodiniaceae genera that associate with *O. faveolata* may have even longer genomes, with *Durusdinium* and *Cladocopium* having genome sizes from 670 to over 1,000 Mb, respectively [[12, 13]](https://paperpile.com/c/wkf7pu/jx8i9+6Nwff). In contrast, the average genome size of cultivated coral-associated prokaryotes is 4.2 Mb [[14]](https://paperpile.com/c/wkf7pu/UxRPD). The disparity between genome sizes – specifically a difference of two to three orders of magnitude – results in a bias of metagenomic reads in favor of the coral host and Symbiodiniaceae, thereby complicating studies of the functional contributions of coral-associated prokaryotes to host health. As a result, existing metagenomic studies of SCTLD often report elevated numbers of bacterial and archaeal genes in diseased tissues, which contain a shifted microbial community and potentially a reduced signal from the tissue-associated algal symbionts [[15, 16]](https://paperpile.com/c/wkf7pu/VSJPK+ReHcw). To focus on the prokaryotic component of apparently healthy coral, some studies use additional filtration of tissues to enrich microorganisms [[17, 18]](https://paperpile.com/c/wkf7pu/tA1x8+CByYE). Alternatively, sequencing deeply to recover a large proportion of microbial reads is essential when host contamination is expected to be above 90% [[19]](https://paperpile.com/c/wkf7pu/vVAt3). Since we are interested in signatures of resilience and resistance to disease, targeting apparently healthy tissue before it is actively degraded is key. Additionally, using deep sequencing enables the recovery of the prokaryotic component of the coral holobiont.

### DNA Extraction and Library preparation

Samples were randomized into batches of eight for DNA extraction. For DNA extraction, coral cores were thawed on ice. Once thawed, the coral tissue was scraped from cores using flame-sterilized scalpels and forceps over a sterile Petri dish. The scraping involved collecting a portion of the coral surface mucus as well as 1-2 polyps into sterile tubes containing 1mL of phosphate-buffered saline with ethylenediaminetetraacetic acid (PBSE). The PBSE solution was created following directions in a previous *Lophelia* coral metagenome extraction protocol [[20]](https://paperpile.com/c/wkf7pu/JDsNo). A sterile micropestle was then used to mechanically break up the tissue and create a slurry. The resulting tissue slurry was then added to the Qiagen QIAamp DNA Microbiome Kit (Qiagen, Germantown, MD, USA) to preferentially extract the microbial DNA instead of host DNA according to the manufacturer’s protocol. Reagent blanks were generated for each batch of DNA extractions wherein the blanks were processed alongside experimental samples, without the addition of coral biomass. Nucleic acid extracts were quantified with a DeNovix high-sensitivity dsDNA fluorescence assay to verify extraction success.

The extracted DNA was then submitted to the University of Florida’s Interdisciplinary Center for Biotechnology Research NextGen DNA Sequencing core (RRID:SCR_019152) for library preparation and sequencing. The NEBNext® Ultra™ II DNA Library Prep Kit for Illumina (New England Biolabs, Ipswich, MA, USA) was used to prepare the sample libraries with the NEBNext® Multiplex Oligos for Illumina® (96 Unique Dual Index Primer Pairs; New England Biolabs, Ipswich, MA, USA). Prepared sample libraries were then sequenced over four lanes on an Illumina NovaSeq 6000 (Illumina, San Diego, CA, USA) in a 150-bp paired-end format. Raw fastq files are openly available through the NCBI Sequence Read Archive under accession number PRJNA925892.

### Reagent Blank Analysis

Due to low biomass, we did not sequence the reagent blank samples with the metagenomic sequencing on the Illumina NovaSeq. Instead, we subjected the four blanks to 16S rRNA gene sequencing. The V4 hypervariable region of bacterial and archaeal 16S rRNA genes was amplified with the Earth Microbiome Protocol [[21]](https://paperpile.com/c/wkf7pu/keU0n) using the 515F [[22]](https://paperpile.com/c/wkf7pu/P8CqU) and 806R [[23]](https://paperpile.com/c/wkf7pu/8eqDO) primers, as previously described [[24]](https://paperpile.com/c/wkf7pu/v3Z7Y). Amplicon libraries were submitted to the University of Florida’s Interdisciplinary Center for Biotechnology Research NextGen DNA Sequencing core (RRID:SCR_019152) for sequencing on an Illumina MiSeq with the 2 x 150 base pair (bp) v2 cycle format. Following sequencing, we used cutadapt v3.4 [[25]](https://paperpile.com/c/wkf7pu/1gOcR) followed by dada2 (v1.30.0) to parse the fastq files into amplicon sequence variants (ASV) [[26]](https://paperpile.com/c/wkf7pu/tb45T). We used the following parameters for filterAndTrim: truncLen=c(145,145), maxN=0, maxEE=c(2,2), truncQ=2, and rm.phix=TRUE. Following filtering, learning error rates, and merging of paired reads, we removed chimeric sequences and taxonomically identified each ASV to the species level with the SILVA database v.138.2 [[27]](https://paperpile.com/c/wkf7pu/JZ8bT). The resultant table of ASV counts and taxonomy are available in Table S7.

### Bioinformatics

An overview of the bioinformatics pipeline we employed to target bacteria and archaea within the coral holobiont is outlined in Figure S1. We assessed the quality of raw reads using FastQC v.0.11.7, then trimmed and filtered the raw reads using trimmomatic v.0.39 (Bolger, Lohse, and Usadel 2014). The parameters included LEADING:3; TRAILING:3; SLIDINGWINDOW:4:20; MINLEN:50. To remove host and endosymbiont DNA and begin selecting for prokaryotic genomic content, we used Bowtie2 v.2.4.2 with parameters: --un-conc-gz to retain unaligned reads [[28]](https://paperpile.com/c/wkf7pu/lSLpl) to map trimmed reads against the following genomes: the *O. faveolata* genome (NCBI Assembly Accession ID GCA_001896105.1), *Breviolum minutum* (NCBI Assembly Accession ID GCA_000507305.1; [[11]](https://paperpile.com/c/wkf7pu/NhVcf)), *Symbiodinium* sp. Clade C Y103 genome (*Cladocopium*; NCBI Assembly Accession ID: GCA_003297045.1; [[29]](https://paperpile.com/c/wkf7pu/ldzFs)), and *Durusdinium sp.* (NCBI Project ID: [PRJDB10306](https://www.ncbi.nlm.nih.gov/bioproject/?term=prjdb10306)). The number of reads retained after this initial quality filtering are listed in Table S2. We assembled the host/endosymbiont-cleaned reads from each of the 41 samples individually into contigs using MegaHit v.1.1.3, employing the –metalarge parameter that is optimized for assembling large, complex datasets [[30]](https://paperpile.com/c/wkf7pu/mPLnt). This resulted in a metagenome assembly for each *O. faveolata* colony*.* These metagenome assemblies are available at Zenodo DOI 10.5281/zenodo.11493775 [[31]](https://paperpile.com/c/wkf7pu/Vq8Hs). These metagenome assemblies were further assessed using QUAST v.5.2.0 [[32]](https://paperpile.com/c/wkf7pu/fbVcu) to determine statistics such as total length, largest contig, and number of contigs (Table S2).

To evaluate the broad prokaryotic and eukaryotic content of the metagenome assemblies, we used EukRep v.0.6.7 [[33]](https://paperpile.com/c/wkf7pu/7x1xH) with the parameters --min 1000 and --prokarya to keep only contigs over 1000 bp and to parse prokaryotic versus eukaryotic contigs. We then counted the number of contigs in each group and used QUAST v.5.2.0 using default parameters to generate summary statistics on the prokaryotic portion of the metagenome assemblies (Table S2).

Following host and symbiont removal, we noted that prokaryotic reads were only 9.66 ± 4.31% of the total assembly length, so we further enriched the prokaryotic component of the host/endosymbiont-cleaned reads prior to generating a co-assembly (Table S2). We used Bowtie2 v.2.4.2 [[28]](https://paperpile.com/c/wkf7pu/lSLpl) to map the host/endosymbiont-cleaned reads to the eukaryotic portion of the metagenome assembly generated by EukRep. We first used bowtie2-build to create an index of the eukaryotic portion of the metagenome assembly using default parameters. Then, we used bowtie2 to map the reads to the eukaryotic indices and retain reads that did not map with the flags --un-conc-gz, --phred33, and -q. which we refer to as eukaryote-removed reads.

In order to recover longer contigs for greater bacterial functional annotation, maintain rare taxa, and to enable cross-sample comparison, we generated a co-assembly of all 41 coral samples. Previous co-assemblies in marine environments have created separate assemblies for different ocean regions and depths [[34]](https://paperpile.com/c/wkf7pu/iPgxB). The coral microbiome is known to differ by region in Florida’s reefs [[35, 36]](https://paperpile.com/c/wkf7pu/PV6Sx+LHyI1). As a result, we investigated if we should generate two separate co-assemblies (one per reef) or one co-assembly combining both reefs. Prior to generating a co-assembly, we used sourmash v.4.5.0 [[37]](https://paperpile.com/c/wkf7pu/Js0aS) to generate hash sketches and investigate the broad read-level similarity of eukaryote-removed reads across the samples to verify samples would co-assemble well. Specifically, we used sourmash sketch dna (parameters k=31), followed by sourmash compare, followed by sourmash plot (parameters --labels). Since the hash sketches did not differ or separate by reef (Looe Key versus Sand Key), we concluded the genomic content was similar and combined them into a co-assembly. To generate the co-assembly, we used MegaHit v.1.1.3 [[30]](https://paperpile.com/c/wkf7pu/mPLnt) with parameters --min-contig-len 1000, -t 100, --presets meta-large. The meta-large preset is for large and complex metagenomes, which we expected for coral metagenomes. We evaluated the resultant assembly with QUAST v.5.2.0 using default parameters. Despite multiple steps of eukaryotic read removal, we anticipated there might still be eukaryotic content. Therefore we used EukRep v.0.6.7 again with the same parameters as before to assess the relative proportion of the co-assembly that was prokaryotic and eukaryotic. We used the prokaryotic portion of the co-assembly reflecting 41 *O. faveolata* corals for subsequent annotation and analysis.

To predict protein-coding genes, we used Prodigal v.2.6.3 [[38]](https://paperpile.com/c/wkf7pu/4gJyw) with the parameters “-f gff” to output a gff file and “-p meta” because the input file was a metagenome. We additionally generated a nucleotide fasta of the predicted protein coding genes (-d flag) for downstream abundance calculation. We annotated the predicted genes (amino acid fasta) with MMseqs2 v.2/14, parameters --search-type 1, --tax-lineage 1, --threads 50, --compressed 1), employing the GTDB database v.214.1 to generate taxonomic annotations [[39]](https://paperpile.com/c/wkf7pu/mDi2k). For functional annotations, we used the KEGG “Prokaryotes + Viruses” database using the web-based GhostKOALA tool to annotate the prokaryote predicted genes (amino acid fasta) [[40]](https://paperpile.com/c/wkf7pu/HICy8). We quantified the abundance of predicted genes from the prokaryote co-assembly using Salmon v.1.10.1 [[41]](https://paperpile.com/c/wkf7pu/rLd5K). We first indexed the nucleotide fasta of the predicted genes (salmon index, parameters -k 31), then mapped the eukaryote-removed reads to the index using salmon quant (parameters --meta, --libType A, -p 36). We saved the “NumReads” column, which represents the number of reads generated by Salmon based on the length and relative abundance of the gene. We saved these values as integers from the Salmon output for each coral sample and aggregated it into one abundance table. The resultant table represented read counts for each predicted gene in the prokaryotic co-assembly for each coral sample, and was used for downstream analyses. The predicted proteins, annotations, and abundance table are publicly available at Zenodo DOI 10.5281/zenodo.11493775 [[31]](https://paperpile.com/c/wkf7pu/Vq8Hs).

We used the resistance gene identifier (RGI) v.6.0.2 to annotate the predicted genes from the prokaryote co-assembly (amino acid fasta) using the Comprehensive Antibiotic Resistance Database (CARD) database, with the parameters: --clean --include_loose -t protein -n 20 [[42]](https://paperpile.com/c/wkf7pu/ixbqF). We used the “--include_loose” parameter to include “loose” hits to the database in addition to strict and perfect hits. This parameter is better for detecting new or distant homologs of antimicrobial resistance genes, and since this coral microbiome dataset is environmental, we expected this parameter to be better suited for our dataset, as we would not expect many clinically-relevant antimicrobial resistance genes. To identify BGCs, we used antiSMASH v.7.0.0 on the prokaryotic co-assembly generated by EukRep (nucleotide fasta containing contigs), using the parameters: --taxon bacteria --tigrfam --pfam2go --cc-mibig --rre --genefinding-tool prodigal-m --cpus 25 [[43]](https://paperpile.com/c/wkf7pu/Ej1sE).

### Statistical analyses

For statistical tests, we used the read counts (“NumReads” from Salmon as integers) of each predicted gene across coral samples (247,254 genes). We analyzed the functional microbial beta diversity using Aitchison distance to account for the compositional structure of the sequencing data [[44]](https://paperpile.com/c/wkf7pu/QRL60). To do this we applied a centered log-ratio (CLR) transformation on the abundance-filtered read counts using the microbiome package v.1.24.0 in R v.4.3.2 [[45]](https://paperpile.com/c/wkf7pu/JIWuc), which incorporates a pseudocount of min(relative abundance)/2 to relative abundances of zero prior to conducting the CLR transformation. We then conducted the ordination and principal components analysis which uses Euclidean distance with phyloseq v.1.46.0 [[46]](https://paperpile.com/c/wkf7pu/vbJRt). To test our hypotheses and examine the extent to which colony-specific covariates (coral genotype, fate, disease condition at sampling, Symbiodiniaceae composition, and reef location) explained variability in the functional microbiome, we used the adonis2 function [[47]](https://paperpile.com/c/wkf7pu/IkEZg) in vegan v.2.6.6.1 to conduct a PERMANOVA. We used the format adonis2(formula = dissimilaritymatrix ~ variable_of_interest, data = metadata, permutations = 999). We first investigated coral genotype-based changes to the functional microbiome to justify treating coral genotypic clones [[5]](https://paperpile.com/c/wkf7pu/XRjV8) as individuals or aggregating their functional microbiomes for further analyses. Based on the results, we chose to treat the genotypic clones as separate individuals for all subsequent analyses (Figure S2a, Supplementary Results). To investigate how dispersion of the prokaryotic microbiome changed with colony fate, condition at sampling, or Symbiodiniaceae community composition, we calculated the distance to centroid in the principal components analysis using the betadisper function in vegan v.2.6.6.1. We hypothesized dispersion would increase in diseased colonies [[48]](https://paperpile.com/c/wkf7pu/3ksHO). Then, based on the normal distribution of the distances, we conducted a Kruskal-Wallis or ANOVA test followed by either a Wilcoxon or Tukey post-hoc test to investigate differences across groupings. For the pairwise wilcoxon test, we used Benjamini-Hochberg false discovery rate correction in the pairwise.wilcox.test function in R and for the Tukey post-hoc test we used the built-in p-value adjustment in the TukeyHSD function in R.

To identify genes that may be involved in disease resistance, we conducted a differential abundance test in corncob between unaffected corals (baseline) and the other colony fates: recovered, will be diseased, and diseased. To glean biological information from the differential abundance analysis, we conducted the differential abundance test only on the 31,695 genes annotated by ghostKOALA. We used corncob v.0.4.1 [[49]](https://paperpile.com/c/wkf7pu/UIGKC) to test the hypotheses that microbial functional genes differed in abundance between apparently healthy tissue in corals from unaffected fates and either recovered, will be diseased, or diseased coral fates. We controlled for the composition of Symbiodiniaceae as that significantly structured the functional microbial community. Corncob modeled relative abundances from the input count data, and differential abundance was modeled as a linear function of coral fate. We controlled for the effect of fate and Symbiodiniaceae on differential variance/overdispersion in the corncob model setup. Significant differences in abundance were evaluated using the parametric Wald test, with a Benjamini-Hochberg false discovery rate correction of 0.05 to account for multiple comparisons. Coefficients including 95% confidence intervals were plotted to display differentially abundant genes. To plot the data, the KEGG annotations were retrieved for each KO ID using the KEGG API in R and parsed into different levels.

To test whether repeated amoxicillin treatments impacted the richness and diversity of beta-lactamase genes, we subset genes with the string “beta-lactamase” in the AMR Gene Family annotation, and used the package breakaway v.4.8.4 to estimate the richness of beta-lactamase genes within each *O. faveolata* colony [[50]](https://paperpile.com/c/wkf7pu/ITGhP). We then tested for the effect of amoxicillin treatments and recent treatment history on estimated beta-lactamase gene richness using the betta function in the breakaway package [[51]](https://paperpile.com/c/wkf7pu/m1CNO). We additionally tested for the effect of these variables on the beta diversity of beta-lactamase genes by generating a PCA and PERMANOVA test using methods described above. The variables we tested for their effect on richness and beta diversity included both raw numbers of total amoxicillin treatments prior to sampling (continuous variable) and the number of treatments of amoxicillin scaled to the estimated live tissue area in meters squared (continuous). Additionally, to test whether treatment history affected diversity of beta-lactamases, we used a categorical variable indicating a colony was never treated, treated more than two months prior to sampling, or treated within two months prior to sampling.

We inferred the potential activity or function of each BGC by using matches to the Minimum Information about a Biosynthetic Gene cluster (MIBiG) database. AntiSMASH reported matches of any portion of a BGC to entries within the MIBiG database. We chose the match with the highest similarity score and reported the MIBiG accession number, similarity score, compound, source organism, and relevant citation from the MIBiG database in Table S3. We used experimental data and information from the MIBiG reference or in some cases additional literature searches to identify the activity or function of the reported compound. The similarity scores were low for the majority of MIBiG matches, which we expected given the complex nature of coral and environmental samples. Therefore, it is likely that the close matches from the coral holobiont may not be the exact compounds referenced in MIBiG. Instead, we used these matches to infer the potential function or activity (siderophore, antimicrobial, toxin, etc) for the BGC. To track how each BGC was annotated and assigned the inferred activity or function, we generated an alluvial plot.

To test whether BGCs with roles related to coral disease resilience were differentially prevalent across colony fates, we used a Fisher’s Exact Test incorporating a Monte-Carlo simulation to generate a p-value, followed by a Benjamini-Hochberg multiple test correction in R. Following manual curation of antiSMASH and MIBiG results, we used a subset of the BGCs which we hypothesized would play a role in coral disease resilience, including gene clusters annotated as “antimicrobial”, “antimicrobial, cytotoxic”, “antimicrobial precursor”, “antimicrobial defense”, “antibiotic resistance”, “cytotoxic”, “phytotoxic”, “toxin”, or “virulence factor”. CoverM v.0.6.1 was used to measure mean coverage using coverm contig with the parameters “--methods mean” and “--min-covered-fraction 10” from the eukaryote removed reads [[52]](https://paperpile.com/c/wkf7pu/9pL5H) and then converted coverage to presence/absence for statistical analysis. We chose to include --min-covered-fraction so that at least 10% of the contig length was mapped, and if less than 10% was covered, the mean coverage was reported as zero. This was included as a way to reduce spurious alignments and false positives [[52]](https://paperpile.com/c/wkf7pu/9pL5H). Additionally, mean coverage represents the average number of bases aligned exactly over the entire contig, excluding the ends. Further, to be conservative we chose to not use an abundance-based metric and instead chose to represent the coverage as either present or absent. We chose a positive average coverage (non-zero mean coverage over at least 10% of the contig) as representing presence and zero as representing absence.

# Results and Discussion

### Clone analysis

Previous work sequenced the genotypes of all colonies included in the analysis [[5]](https://paperpile.com/c/wkf7pu/XRjV8). Of the 41 colonies, 33 were identified as unique genotypes, with four sets of clones (Figure S2). Three sets of two clones were at Looe Key and one set of three was at Sand Key. Due to non-independence of the colonies, we investigated how clone impacted functional microbiome beta diversity. We determined that microbiome beta diversity did not change due to clone (Figure S2a, Table S4). In one case of a set of clones at Looe Key (colony ID 699 and N59), one colony was actively diseased (699) and the other was apparently healthy (N59) at the time of sampling and ultimately was unaffected for the duration of the study. Additionally, for clone sets A and C (Figure S2a), the functional microbiome beta diversity was much greater than that between individuals that were not clones. This led us to conclude that despite host genotype, environmental and other differences not measured here led to divergent outcomes and microbial dissimilarity, and as a result these colonies were not treated as clones in the study. Further justification for treating these clones came from PERMANOVA-based statistics. We controlled for clone set on PERMANOVA tests by setting permutation blocks in the adonis2 function in vegan. For all tested parameters, the p-value generated the same overall result, or an even smaller p-value for significant results generated when not controlling for clone. As a result, we chose to use the original data without controlling for clones as we determined clones are not a significant driver of functional microbiome beta diversity in these coral colonies.

### Antibiotic Resistance Gene Diversity

The coral microbiome encoded over 200,000 ARGs, of which over 50,000 were beta-lactamase genes (Figure S7, Table S7). These ARGs were present across *O. faveolata* corals. Most ARGs encoded efflux or inactivation mechanisms of resistance, along with 13 other mechanisms of resistance (Figure S7a). These genes fell into 437 different ARG families, of which the top 15 comprised between 37 - 52% of the abundance of all ARGs (Figure S7b). Within the beta-lactamases, the most abundant families were YEM, IMP, SGM, and OXA beta-lactamases, and the top 15 beta-lactamase gene families made 24 - 46% of all beta-lactamase gene counts in colonies (Figure S7c).

### Blank Analysis

We generated 16S rRNA gene-based analyses of four DNA extraction reagent blanks to identify potential contaminants. Of the four blanks, two contained about 17,000 reads, while the other two contained about 1,200 reads (Figure S8, Table S7). The most abundant reagent blank sequences were assigned to the following genera: *Acetobacter, Brucella, Corynebacterium, Lacticaseibacillus, Lentilactobacilus, Liquorilactobacillus, Niallia, Novosphingobium, Peribacillus, Scheiferilactobacillus, Staphylococcus, and Zanthobacter* (Figure S8). A complete list of taxa in the blanks is in Table S7.

One challenge in this study was the lack of reagent blanks that matched the full processing steps of the metagenome samples. Reagent blanks from the DNA extraction process were subjected to PCR amplification of the 16S rRNA gene for taxonomic microbiome analysis, which resulted in 208 ASVs, that substituted for reagent blanks that included shotgun metagenomic library preparation. We reasoned that library preparation would be unsuccessful with the low- to no-biomass reagent blanks and would be rejected by the sequencing center. In hindsight, this could have been overcome with the addition of mock community DNA to reagent blanks prior to sequencing, and this is a tactic recommended for future studies. We found some taxa in the results that overlapped with those found in the DNA blanks. For example, we identified one *Acetobacter* and *Corynebacterium* gene each within our differentially abundant analysis, and these were also in three or two out of the four DNA blanks, respectively. While *Acetobacter* is a genus of acetic acid bacteria associated with fuel tank corrosion, it has also been isolated from soft corals [[53, 54]](https://paperpile.com/c/wkf7pu/swqao+RVC8x). Similarly, *Corynebacterium* has been isolated from a hard *Fungia* coral, as well as associated with human skin infections [[55, 56]](https://paperpile.com/c/wkf7pu/qoEwA+c6Gh5). Of the top 20 most abundant taxa within the DNA blanks, *Staphylococcus* was represented most within the differentially abundant functional genes. *Staphylococcus epidermis* is a common skin bacterium and pathogen [[57]](https://paperpile.com/c/wkf7pu/iq2nu). *Staphylococcus* is also a coral opportunistic pathogen and is commonly found in the coral microbiome [[58–60]](https://paperpile.com/c/wkf7pu/JvJ6B+G8BlX+ylHjh). While some overlap exists, there were also some taxa reassuringly absent from the DNA blanks, such as *Cutibacterium* (formerly *Propionibacterium*). Many contigs classified as *Cutibacterium,* and one BGC with a perfect similarity score matched a cutimycin gene cluster from *Cutibacterium acnes.* While *Cutibacterium acnes* is a skin commensal and opportunistic pathogen, it has also been found in coral metagenome-assembled genomes, and even been considered a potential endosymbiont [[17, 61, 62]](https://paperpile.com/c/wkf7pu/b1f0m+tA1x8+yMjEu). Overall, while there was some overlap between the taxa recovered in the DNA blanks, we did not remove them from analysis given the different methodological approaches and associated bias (i.e. PCR amplification with 16S rRNA methods) as well as due to prior research linking similar genera with marine and coral habitats. In addition, sequencing errors resulting in sample “cross-talk” where sequence reads are misbarcoded is also a potential issue with the Illumina sequencing platform [[63]](https://paperpile.com/c/wkf7pu/ybtTl), thus taxa detected in the reagent blanks may actually be sourced from true samples. In the future, incorporating both mock community DNA and reagent blanks spiked with mock community DNA in shotgun metagenome sequencing efforts will improve our assessment of potential contamination from cross-talk, kit reagents, or users [[63]](https://paperpile.com/c/wkf7pu/ybtTl).

# References

1.	[Neely KL, Shea CP, Macaulay KA, Hower EK, Dobler MA. Short- and long-term effectiveness of coral disease treatments. *Front Mar Sci* 2021; **8**.](http://paperpile.com/b/wkf7pu/otWIW)

2.	[Neely KL, Macaulay KA, Hower EK, Dobler MA. Effectiveness of topical antibiotics in treating corals affected by Stony Coral Tissue Loss Disease. *PeerJ* 2020; **8**: e9289.](http://paperpile.com/b/wkf7pu/K9gHF)

3.	[Neely KL, Nowicki RJ, Dobler MA, Toth KA, Macaulay KA, Gallagher SM. Survival and reinfection rates of SCTLD-affected corals treated in situ with amoxicillin. *Ecology* . 2025. bioRxiv.](http://paperpile.com/b/wkf7pu/RrBv3)

4.	[Brian K. Walker, Greta S. Aeby, Andrew C. Baker, Samantha Buckley, Neha Garg, Aine Hawthorne, Julie L. Meyer, Karen Neely, Hunter Noren, Valerie J. Paul, Reagan Sharkey, Nikki Traylor-Knowles, Josh D. Voss, David R. Whitall, Gareth J. Williams, Cheryl M. Woodley and Thierry Work. SCTLD resistance research consortium summary report through May 2023. 2023. Florida Department of Environmental Protection.](http://paperpile.com/b/wkf7pu/YCJ0L)

5.	[Klein AM, Sturm AB, Eckert RJ, Walker BK, Neely KL, Voss JD. Algal symbiont genera but not coral host genotypes correlate to stony coral tissue loss disease susceptibility among Orbicella faveolata colonies in South Florida. *Front Mar Sci* 2024; **11**.](http://paperpile.com/b/wkf7pu/XRjV8)

6.	[Cunning R, Silverstein RN, Baker AC. Investigating the causes and consequences of symbiont shuffling in a multi-partner reef coral symbiosis under environmental change. *Proc Biol Sci* 2015; **282**: 20141725.](http://paperpile.com/b/wkf7pu/5BfNg)

7.	[Wegley L, Edwards R, Rodriguez-Brito B, Liu H, Rohwer F. Metagenomic analysis of the microbial community associated with the coral Porites astreoides. *Environ Microbiol* 2007; **9**: 2707–2719.](http://paperpile.com/b/wkf7pu/0kmSF)

8.	[Vega Thurber R, Willner-Hall D, Rodriguez-Mueller B, Desnues C, Edwards RA, Angly F, et al. Metagenomic analysis of stressed coral holobionts. *Environ Microbiol* 2009; **11**: 2148–2163.](http://paperpile.com/b/wkf7pu/dLQ3l)

9.	[Young BD, Williamson OM, Kron NS, Andrade Rodriguez N, Isma LM, MacKnight NJ, et al. Annotated genome and transcriptome of the endangered Caribbean mountainous star coral (Orbicella faveolata) using PacBio long-read sequencing. *BMC Genomics* 2024; **25**: 226.](http://paperpile.com/b/wkf7pu/L8Pid)

10.	[Prada C, Hanna B, Budd AF, Woodley CM, Schmutz J, Grimwood J, et al. Empty niches after extinctions increase population sizes of modern corals. *Curr Biol* 2016; **26**: 3190–3194.](http://paperpile.com/b/wkf7pu/3W5F9)

11.	[Shoguchi E, Shinzato C, Kawashima T, Gyoja F, Mungpakdee S, Koyanagi R, et al. Draft assembly of the Symbiodinium minutum nuclear genome reveals dinoflagellate gene structure. *Curr Biol* 2013; **23**: 1399–1408.](http://paperpile.com/b/wkf7pu/NhVcf)

12.	[Shoguchi E, Beedessee G, Hisata K, Tada I, Narisoko H, Satoh N, et al. A new dinoflagellate genome illuminates a conserved gene cluster involved in sunscreen biosynthesis. *Genome Biol Evol* 2021; **13**.](http://paperpile.com/b/wkf7pu/jx8i9)

13.	[Liu H, Stephens TG, González-Pech RA, Beltran VH, Lapeyre B, Bongaerts P, et al. Symbiodinium genomes reveal adaptive evolution of functions related to coral-dinoflagellate symbiosis. *Commun Biol* 2018; **1**: 95.](http://paperpile.com/b/wkf7pu/6Nwff)

14.	[Li J, Zou Y, Yang J, Li Q, Bourne DG, Sweet M, et al. Cultured bacteria provide insight into the functional potential of the coral-associated microbiome. *mSystems* 2022; **7**: e0032722.](http://paperpile.com/b/wkf7pu/UxRPD)

15.	[Heinz JM, Lu J, Huebner LK, Salzberg SL, Sommer M, Rosales SM. Novel metagenomics analysis of stony coral tissue loss disease. *G3* 2024; **14**.](http://paperpile.com/b/wkf7pu/VSJPK)

16.	[Rosales SM, Huebner LK, Clark AS, McMinds R, Ruzicka RR, Muller EM. Bacterial metabolic potential and micro-eukaryotes enriched in stony coral tissue loss disease lesions. *Frontiers in Marine Science* 2022; **8**: 776859.](http://paperpile.com/b/wkf7pu/ReHcw)

17.	[Messer LF, Bourne DG, Robbins SJ, Clay M, Bell SC, McIlroy SJ, et al. A genome-centric view of the role of the Acropora kenti microbiome in coral health and resilience. *Nat Commun* 2024; **15**: 2902.](http://paperpile.com/b/wkf7pu/tA1x8)

18.	[Robbins SJ, Singleton CM, Chan CX, Messer LF, Geers AU, Ying H, et al. A genomic view of the reef-building coral Porites lutea and its microbial symbionts. *Nat Microbiol* 2019.](http://paperpile.com/b/wkf7pu/CByYE)

19.	[Pereira-Marques J, Hout A, Ferreira RM, Weber M, Pinto-Ribeiro I, van Doorn L-J, et al. Impact of host DNA and sequencing depth on the taxonomic resolution of whole metagenome sequencing for microbiome analysis. *Front Microbiol* 2019; **10**: 1277.](http://paperpile.com/b/wkf7pu/vVAt3)

20.	[Galkiewicz J, Vega-Thurber R, Kellogg C. Lophelia metagenome extraction protocol. 2011.](http://paperpile.com/b/wkf7pu/JDsNo)

21.	[Caporaso JG, Lauber CL, Walters WA, Berg-Lyons D, Huntley J, Fierer N, et al. Ultra-high-throughput microbial community analysis on the Illumina HiSeq and MiSeq platforms. *ISME J* 2012; **6**: 1621–1624.](http://paperpile.com/b/wkf7pu/keU0n)

22.	[Parada AE, Needham DM, Fuhrman JA. Every base matters: assessing small subunit rRNA primers for marine microbiomes with mock communities, time series and global field samples. *Environ Microbiol* 2016; **18**: 1403–1414.](http://paperpile.com/b/wkf7pu/P8CqU)

23.	[Apprill A, McNally S, Parsons R, Weber L. Minor revision to V4 region SSU rRNA 806R gene primer greatly increases detection of SAR11 bacterioplankton. *Aquat Microb Ecol* 2015; **75**: 129–137.](http://paperpile.com/b/wkf7pu/8eqDO)

24.	[Meyer JL, Castellanos-Gell J, Aeby GS, Häse CC, Ushijima B, Paul VJ. Microbial community shifts associated with the ongoing stony coral tissue loss disease outbreak on the Florida Reef Tract. *Front Microbiol* 2019; **10**: 2244.](http://paperpile.com/b/wkf7pu/v3Z7Y)

25.	[Martin M. Cutadapt removes adapter sequences from high-throughput sequencing reads. *EMBnet J* 2011; **17**: 10.](http://paperpile.com/b/wkf7pu/1gOcR)

26.	[Callahan BJ, McMurdie PJ, Rosen MJ, Han AW, Johnson AJA, Holmes SP. DADA2: High-resolution sample inference from Illumina amplicon data. *Nat Methods* 2016; **13**: 581–583.](http://paperpile.com/b/wkf7pu/tb45T)

27.	[Yilmaz P, Parfrey LW, Yarza P, Gerken J, Pruesse E, Quast C, et al. The SILVA and ‘All-species Living Tree Project (LTP)’ taxonomic frameworks. *Nucleic Acids Res* 2014; **42**: D643–8.](http://paperpile.com/b/wkf7pu/JZ8bT)

28.	[Langmead B, Salzberg SL. Fast gapped-read alignment with Bowtie 2. *Nat Methods* 2012; **9**: 357–359.](http://paperpile.com/b/wkf7pu/lSLpl)

29.	[Shoguchi E, Beedessee G, Tada I, Hisata K, Kawashima T, Takeuchi T, et al. Two divergent Symbiodinium genomes reveal conservation of a gene cluster for sunscreen biosynthesis and recently lost genes. *BMC Genomics* 2018; **19**: 458.](http://paperpile.com/b/wkf7pu/ldzFs)

30.	[Li D, Liu C-M, Luo R, Sadakane K, Lam T-W. MEGAHIT: an ultra-fast single-node solution for large and complex metagenomics assembly via succinct de Bruijn graph. *Bioinformatics* 2015; **31**: 1674–1676.](http://paperpile.com/b/wkf7pu/mPLnt)

31.	[Becker C, Cauvin A, Meyer J. *Orbicella faveolata* and *O. franksi* coral metagenome assemblies from the Lower Florida Keys region of Florida, USA. 2024. Zenodo.](http://paperpile.com/b/wkf7pu/Vq8Hs)

32.	[Gurevich A, Saveliev V, Vyahhi N, Tesler G. QUAST: quality assessment tool for genome assemblies. *Bioinformatics* 2013; **29**: 1072–1075.](http://paperpile.com/b/wkf7pu/fbVcu)

33.	[West PT, Probst AJ, Grigoriev IV, Thomas BC, Banfield JF. Genome-reconstruction for eukaryotes from complex natural microbial communities. *Genome Res* 2018; **28**: 569–580.](http://paperpile.com/b/wkf7pu/7x1xH)

34.	[Alexander H, Hu SK, Krinos AI, Pachiadaki M, Tully BJ, Neely CJ, et al. Eukaryotic genomes from a global metagenomic data set illuminate trophic modes and biogeography of ocean plankton. *MBio* 2023; e0167623.](http://paperpile.com/b/wkf7pu/iPgxB)

35.	[Becker CC, Weber L, Zgliczynski B, Sullivan C, Sandin S, Muller E, et al. Microorganisms and dissolved metabolites distinguish Florida’s Coral Reef habitats. *PNAS Nexus* 2023; **2**: gad287.](http://paperpile.com/b/wkf7pu/PV6Sx)

36.	[Williams SD, Klinges JG, Zinman S, Clark AS, Bartels E, Villoch Diaz Maurino M, et al. Geographically driven differences in microbiomes of Acropora cervicornis originating from different regions of Florida’s Coral Reef. *PeerJ* 2022; **10**: e13574.](http://paperpile.com/b/wkf7pu/LHyI1)

37.	[Titus Brown C, Irber L. sourmash: a library for MinHash sketching of DNA. *J Open Source Softw* 2016; **1**: 27.](http://paperpile.com/b/wkf7pu/Js0aS)

38.	[Hyatt D, Chen G-L, Locascio PF, Land ML, Larimer FW, Hauser LJ. Prodigal: prokaryotic gene recognition and translation initiation site identification. *BMC Bioinformatics* 2010; **11**: 119.](http://paperpile.com/b/wkf7pu/4gJyw)

39.	[Steinegger M, Söding J. MMseqs2 enables sensitive protein sequence searching for the analysis of massive data sets. *Nat Biotechnol* 2017; **35**: 1026–1028.](http://paperpile.com/b/wkf7pu/mDi2k)

40.	[Kanehisa M, Sato Y, Morishima K. BlastKOALA and GhostKOALA: KEGG tools for functional characterization of genome and metagenome sequences. *J Mol Biol* 2016; **428**: 726–731.](http://paperpile.com/b/wkf7pu/HICy8)

41.	[Patro R, Duggal G, Love MI, Irizarry RA, Kingsford C. Salmon provides fast and bias-aware quantification of transcript expression. *Nat Methods* 2017; **14**: 417–419.](http://paperpile.com/b/wkf7pu/rLd5K)

42.	[Alcock BP, Huynh W, Chalil R, Smith KW, Raphenya AR, Wlodarski MA, et al. CARD 2023: expanded curation, support for machine learning, and resistome prediction at the Comprehensive Antibiotic Resistance Database. *Nucleic Acids Res* 2023; **51**: D690–D699.](http://paperpile.com/b/wkf7pu/ixbqF)

43.	[Blin K, Shaw S, Augustijn HE, Reitz ZL, Biermann F, Alanjary M, et al. antiSMASH 7.0: new and improved predictions for detection, regulation, chemical structures and visualisation. *Nucleic Acids Res* 2023; **51**: W46–W50.](http://paperpile.com/b/wkf7pu/Ej1sE)

44.	[Gloor GB, Macklaim JM, Pawlowsky-Glahn V, Egozcue JJ. Microbiome datasets are compositional: And this is not optional. *Front Microbiol* 2017; **8**: 2224.](http://paperpile.com/b/wkf7pu/QRL60)

45.	[Lahti L, Shetty S. microbiome R package. 2012-2019.](http://paperpile.com/b/wkf7pu/JIWuc)

46.	[McMurdie PJ, Holmes S. phyloseq: An R package for reproducible interactive analysis and graphics of microbiome census data. *PLoS ONE* 2013; **8**: e61217.](http://paperpile.com/b/wkf7pu/vbJRt)

47.	[Oksanen J, Simpson GL, Blanchet FG, Kindt R, Legendre P, Minchin PR, et al. vegan: Community Ecology Package. 2024.](http://paperpile.com/b/wkf7pu/IkEZg)

48.	[Zaneveld JR, McMinds R, Vega Thurber R, Thurber RV. Stress and stability: applying the Anna Karenina principle to animal microbiomes. Nat Microbiol 2: 17121. 2017.](http://paperpile.com/b/wkf7pu/3ksHO)

49.	[Martin BD, Witten D, Willis AD. Modeling microbial abundances and dysbiosis with beta-binomial regression. *Ann Appl Stat* 2020; **14**: 94–115.](http://paperpile.com/b/wkf7pu/UIGKC)

50.	[Willis A, Bunge J. Estimating diversity via frequency ratios: Estimating Diversity via Ratios. *Biometrics* 2015; **71**: 1042–1049.](http://paperpile.com/b/wkf7pu/ITGhP)

51.	[Willis A, Bunge J, Whitman T. Improved detection of changes in species richness in high diversity microbial communities. *J R Stat Soc Ser C Appl Stat* 2017; **66**: 963–977.](http://paperpile.com/b/wkf7pu/m1CNO)

52.	[Aroney STN, Newell RJP, Nissen JN, Camargo AP, Tyson GW, Woodcroft BJ. CoverM: read alignment statistics for metagenomics. *Bioinformatics* 2025; **41**.](http://paperpile.com/b/wkf7pu/9pL5H)

53.	[de Carvalho CCCR. Marine biofilms: A successful microbial strategy with economic implications. *Front Mar Sci* 2018; **5**.](http://paperpile.com/b/wkf7pu/swqao)

54.	[Chen Y, Wang W, Zhou D, Cai B, Zhang M, Qi D, et al. Acetobacter orientalis XJC-C with a high lignocellulosic biomass-degrading ability improves significantly composting efficiency of banana residues by increasing metabolic activity and functional diversity of bacterial community. *Bioresour Technol* 2021; **324**: 124661.](http://paperpile.com/b/wkf7pu/RVC8x)

55.	[Pinto M, Hundi GK, Bhat RM, Bala NK, Dandekeri S, Martis J, et al. Clinical and epidemiological features of coryneform skin infections at a tertiary hospital. *Indian Dermatol Online J* 2016; **7**: 168–173.](http://paperpile.com/b/wkf7pu/qoEwA)

56.	[Ben-Dov E, Ben Yosef DZ, Pavlov V, Kushmaro A. Corynebacterium maris sp. nov., a marine bacterium isolated from the mucus of the coral Fungia granulosa. *Int J Syst Evol Microbiol* 2009; **59**: 2458–2463.](http://paperpile.com/b/wkf7pu/c6Gh5)

57.	[Otto M. Staphylococcus epidermidis--the ‘accidental’ pathogen. *Nat Rev Microbiol* 2009; **7**: 555–567.](http://paperpile.com/b/wkf7pu/iq2nu)

58.	[Reigel AM, Paz-García DA, Hellberg ME. Microbiome of a reef-building coral displays signs of acclimation to a stressful shallow hydrothermal vent habitat. *Front Mar Sci* 2021; **8**.](http://paperpile.com/b/wkf7pu/JvJ6B)

59.	[Divya S, Thinesh T, Seghal Kiran G, Hassan S, Selvin J. Emergence of a multi host biofilm forming opportunistic pathogen Staphylococcus sciuri D26 in coral Favites abdita. *Microb Pathog* 2018; **120**: 204–212.](http://paperpile.com/b/wkf7pu/G8BlX)

60.	[de Castro AP, Araújo SD Jr, Reis AMM, Moura RL, Francini-Filho RB, Pappas G Jr, et al. Bacterial community associated with healthy and diseased reef coral Mussismilia hispida from eastern Brazil. *Microb Ecol* 2010; **59**: 658–667.](http://paperpile.com/b/wkf7pu/ylHjh)

61.	[D Ainsworth T, Krause L, Bridge T, Torda G, Raina J-B, Zakrzewski M, et al. The coral core microbiome identifies rare bacterial taxa as ubiquitous endosymbionts. *ISME J* 2015; **9**: 2261–2274.](http://paperpile.com/b/wkf7pu/b1f0m)

62.	[Mayslich C, Grange PA, Dupin N. Cutibacterium acnes as an opportunistic pathogen: An update of its virulence-associated factors. *Microorganisms* 2021; **9**: 303.](http://paperpile.com/b/wkf7pu/yMjEu)

63.	[Wright ES, Vetsigian KH. Quality filtering of Illumina index reads mitigates sample cross-talk. *BMC Genomics* 2016; **17**: 876.](http://paperpile.com/b/wkf7pu/ybtTl)
